# Interface Wettability Transition‐Driven Drug Release and Dual‐Phase Functionalization in Implant Abutment

**DOI:** 10.1002/advs.75351

**Published:** 2026-04-16

**Authors:** Zhongchao Wang, Liang Shi, MingXia Li, Xiao Han, Jinghan Wang, Guangping Wang, Dan Zou, Bingyang Lu, Liyuan Fan

**Affiliations:** ^1^ Department of Periodontics & Oral Mucosal Diseases The Affiliated Stomatological Hospital Southwest Medical University Luzhou Sichuan China; ^2^ Luzhou Key Laboratory of Oral & Maxillofacial Reconstruction and Regeneration Luzhou Sichuan China; ^3^ Institute of Stomatology Southwest Medical University Luzhou Sichuan China; ^4^ Department of Prosthodontics The Affiliated Stomatological Hospital Southwest Medical University Luzhou Sichuan China; ^5^ Department of Oral Radiology The Affiliated Stomatological Hospital Southwest Medical University Luzhou Sichuan China; ^6^ Institute of Fundamental and Frontier Sciences University of Electronic Science and Technology of China Chengdu P. R. China; ^7^ Department of Orthodontics The Affiliated Stomatological Hospital Southwest Medical University Luzhou Sichuan China; ^8^ School of Comprehensive Health Management Xihua University Chengdu Sichuan Province P. R. China

**Keywords:** anti‐fouling, drug release, implant abutments, interfacial wettability, micro‐environment regulation

## Abstract

Dental implant abutments face dual clinical challenges of early‐stage biological contamination and late‐stage inflammatory microenvironments. To address the issues, this study developed a smart drug‐release coating based on a superhydrophobic‐wettable transition mechanism. The coating on the abutment surface was constructed by hydrophobic micelles self‐assembling on mesoporous silica nanoparticles loaded with berberine. In the early stage, the superhydrophobic surface can effectively resist the adhesion of proteins and bacteria. With protein deposition, this process induced a transition of the superhydrophobic surface to a hydrophilic state, leading to a sigmoidal drug release profile that passively triggered sustained microenvironment regulation in the later stage. In vivo experiments demonstrated that this coating not only had strong immediate antibacterial efficacy (22.5‐fold enhancement) but also significantly reduced inflammation and promoted soft tissue closure. This work provides a sequential functionalization strategy of “early‐antifouling‐enabled late active regulation” for abutments, offering a potential solution to prevent peri‑implant inflammation and soft tissue dehiscence.

## Graphical abstract

A temporally controlled drug release coating is developed for abutments using a superhydrophobic coating self‐assembled on mesoporous silica. This surface provides sequential functionality: initial contamination resistance through non‐wetting, followed by controlled drug release via wettability transition. The coating demonstrates 22.5 times enhanced antibacterial efficacy, anti‐inflammatory, and improved soft tissue integration, offering a reliable research direction for preventing peri‐implant complications.

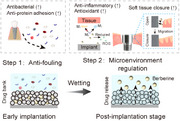

## 1 Introduction

The development of dental implants has greatly improved the treatment of oral diseases, yet the biggest challenges of its application were the immunological rejection and inflammation after implantation [1, 2]. For example, in the early stage of implantation, dental implants are exposed to a complex physiological environment involving saliva, proteins, blood, and various micro‐organisms, which increases the risk of peri‐implant infection [3]. The abutment was the core parts in the implant, because it is exposed in the mouth for a long term and faces a complex environment. After implantation, foreign‐body reactions lead to quick protein and bacteria adsorption on the abutment surface, which facilitates early biofilm formation that is the initial step in the development of infections such as peri‐implantitis [4, 5]. Besides, the biologically contaminated abutment supported the fast channel for bacterial migration to the implant thread, ultimately compromising the implant stability. In addition to the requirements for the anti‐fouling ability of the abutment, it was essential to regulate the peri‐implant microenvironment to deal with the chronic inflammation response and promote soft‐tissue integration [6, 7]. Therefore, developing abutments that can simultaneously prevent early‐stage contamination and regulate late‐stage inflammation proposes a promising direction for advancing dental implant therapy [8].

Recent research has focused on the drug coating and surface modification to enhance the effective sealing of soft tissues and augment the antibacterial properties [9, 10]. However, these functional surfaces lack sequential regulatory ability. For example, conventional drug coatings typically present burst drug release behavior, which leads to a short early‐stage effect, a low drug utilisation rate due to rapid clearance by the blood and saliva. Meanwhile, the modification of the surface usually had incomplete multi‐functions [11, 12]. Although stimulus‐responsive on‐demand release and temporally controlled drug delivery systems have been widely studied [13], these applications to implant abutment remain challenging, because the pharmacokinetic analysis of the effects of multiple drugs was difficult and the oral microenvironment was complex. Delaying the bulk release of drugs post‑implantation helps prevent an initial burst release, thereby enhancing drug utilisation efficiency. Therefore, it was necessary to explore new strategies to meet the therapeutic requirements.

Superhydrophobic (SHPO) surface had attracted considerable attention through the bionic lotus leaf, and it showed that it could effectively prevent surface contamination, such as anti‐icing, dust‐proof, anti‐fouling, and so on [14, 15]. The special property stems from its unique solid–liquid‐gas three‐phase interface, which minimises the solid–liquid contact area. The extremely low solid–liquid contact area limited protein adhesion, which dramatically reduced the adhesion of proteins and thrombus on the surface [16, 17]. However, during the process of contact with biological liquids, proteins inevitably deposit at the three‐phase interface, inducing the interface contact line to slip and triggering a transition in interface state [18]. The superhydrophobic‐hydrophilic transition corresponds to significant surface functions and characteristics. Interestingly, it might be used to delay and control the release of drugs after the interface states changed by the protein adhesion behavior, when the drug was loaded into the SHPO surface [19]. This adaptive mechanism integrates the shift from a superhydrophobic to a hydrophilic state with precise drug release and functional modulation, enabling real‑time responsiveness in the implanted area. By sensing wound severity—reflected in proteins and blood concentration—the system dynamically adjusts its surface wettability to either accelerate or sustain drug release as required. This passive intelligent release model is particularly suitable for the surface coating of implant abutment, and it can adjust the function of the abutment step by step as required. Specifically, during the early post‐implantation phase, the superhydrophobic surface can effectively prevent protein and bacterial adhesion. As the surface state transition triggered by protein deposition progresses, drug release can regulate the peri‐implant microenvironment.

**FIGURE 1:**
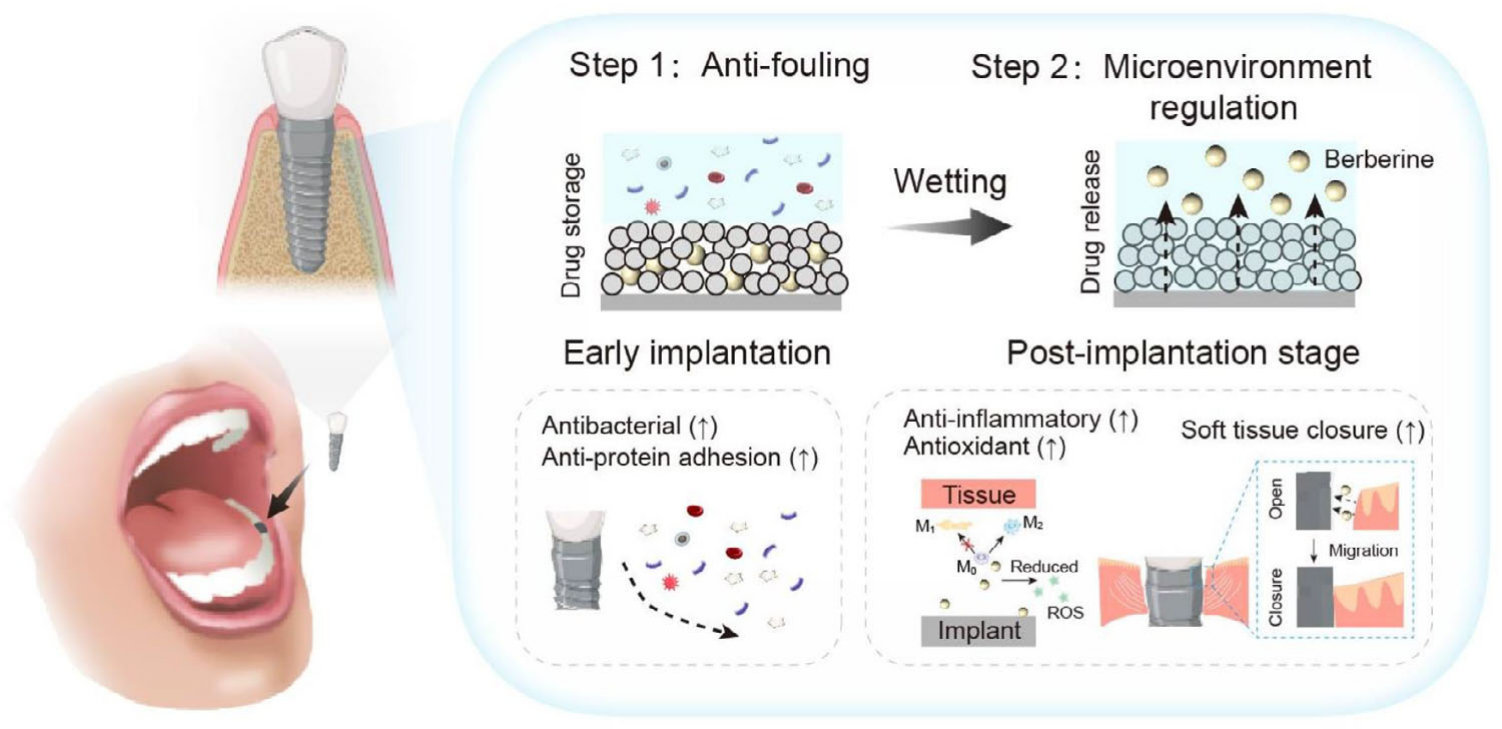
Design of the dual‐phase functionalization with sigmoidal drug release coating in an implant abutment based on interface wettability regulation.

Here, we developed an implant abutment coating that enables sequential biological regulation through a superhydrophobic‐state‐transition‐mediated sigmoidal drug release profile, which achieved both the early biological anti‐fouling and late micro‐environment regulation (Figure 1). The superhydrophobic coating was bonded onto the mesoporous silica nanoparticles (MSNs) surface through self‐assembly, which increased the antibacterial and anti‐protein properties on the surface by more than 12.3‐fold and 8.6‐fold, respectively. And the berberine chloride (Ber) was loaded into the MSNs, providing sustained antibacterial activity after surface wetting and bringing inflammatory regulatory performance to the abutment. In vivo experiments demonstrated that the SHPO‐Ber coating not only showed superior antibacterial (22.5‐fold) and anti‐inflammatory abilities, but also greatly enhanced the soft tissue closure efficacy (compared to pure titanium). We envision that this sigmoidal drug release model, through the regulation of the superhydrophobic surface states, can serve as a development direction to effectively offer suitable sequential drug coatings for dental implant abutment and many other biomedical needs.

## 2 Results

### 2.1 Preparation and Characterization of the SHPO‐Ber Coating

**FIGURE 2:**
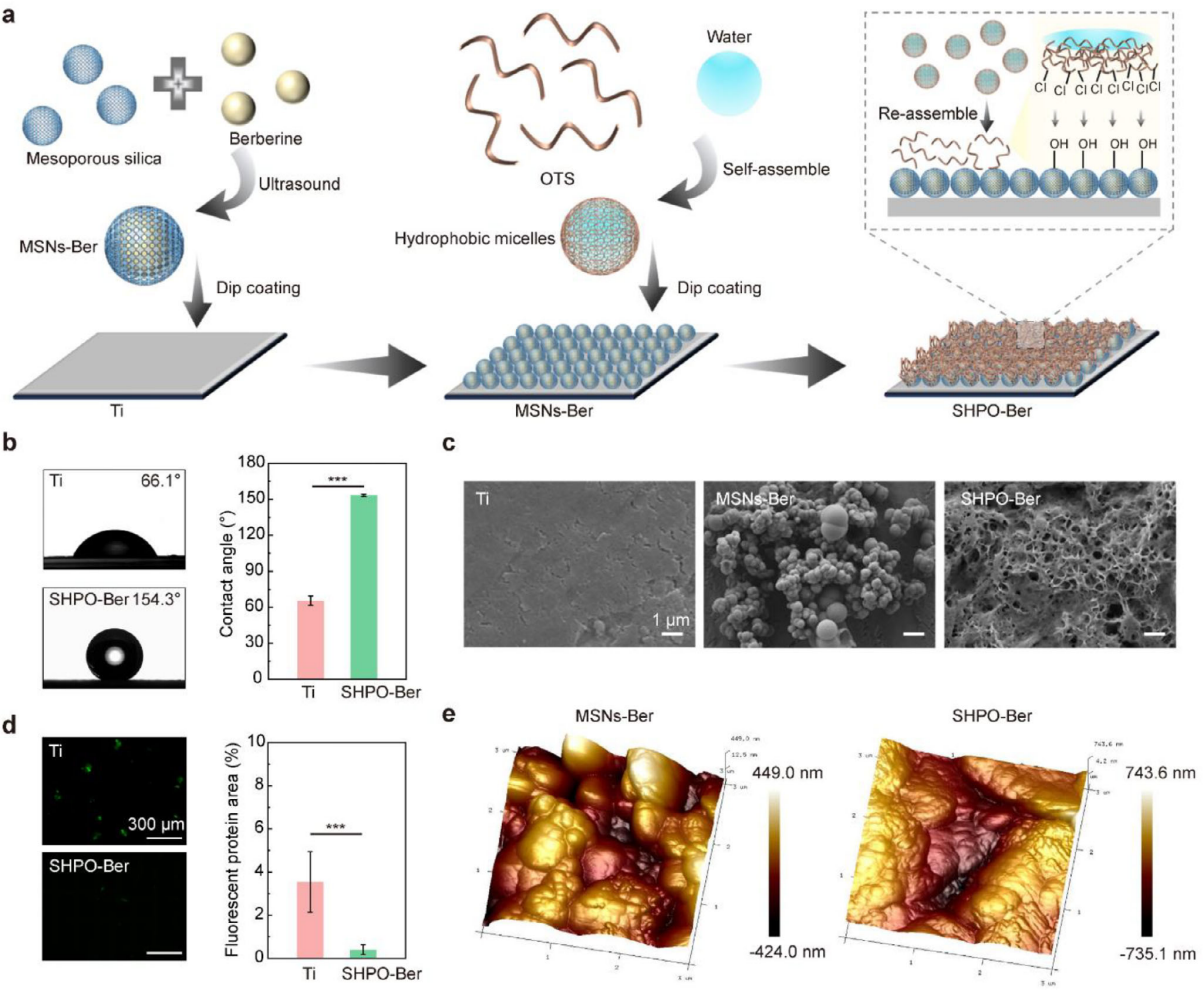
Preparation and characterization of the SHPO‐Ber. (a) Schematic of the sigmoidal drug release SHPO‐Ber preparation. (b) Water contact angle of SHPO‐Ber preparation on Ti. (c) SEM images of SHPO‐Ber preparation process. (d) Images and percentage fluorescence of FITC‐BSA adhesion on the samples at days 1. (e) AFM images of SHPO‐Ber coatings before and after superhydrophobicity. (n = 3, analyzed using paired t test, ^***^ *p* < 0.001).

Berberine chloride (Ber) was loaded into the mesoporous silica nanoparticles (MSNs) via ultrasound; the Ber load rate is approximately 8.87%. Through the SEM‐EDS analysis (Figure S1), the MSNs‐Ber group showed the presence of 0.11% nitrogen (N), whereas no nitrogen was detected in the MSNs group (Table S1). This result showed that Ber was successfully loaded into MSNs, because the N element only existed in Ber, and Ber was easily loaded on the Ti surface with the MSNs deposition (0.225 mg/cm^3^). To get a superhydrophobic (SHPO) surface, the octadecyltrichlorosilane (OTS) was applied to form the hydrophobic micelles by self‐assembly in the presence of water. The hydrophobic micelles still had active chlorine groups, which could react with hydroxyl groups on MSNs as shown in Figure 2a, enabling rapid and stable formation of a superhydrophobic coating [20]. Through the water contact angle measurement (Figure 2b), the water contact angle of SHPO‐Ber was increased to 154.3° from 66.1° (Ti), confirming the successful preparation of superhydrophobic Ber‐loaded coating (SHPO‐Ber). As shown in Figure 2c, SEM imaging revealed that MSNs‐Ber were uniformly dispersed on the Ti surface as expected, and a small amount of nanoparticle agglomeration could provide surface roughness. In the dipping coating process, the hydrophobic micelles reassembled on the MSNs surface, where the active chlorine could be combined with the hydroxyl groups on the surface of silica nanoparticles [21]. After reassembly, the hydrophobic micelles formed a networked structure on the MSNs surface, which effectively protected the MSNs and inhibited burst release of Ber.

The SHPO‐Ber could effectively reduce biological contamination owing to its superhydrophobic capacity, and the low solid–liquid contact area was beneficial for interfacial slippage and desorption of proteins [14]. FITC‐BSA, as a model protein, was used to measure the anti‐fouling performance of SHPO‐Ber [22]. As shown in Figure 2d, it showed that there was only about 0.41% proteins adhered on the SHPO‐Ber surface, while the surface of pure Ti had 3.54% proteins adhesion area, which significantly increased compared with the SHPO‐Ber group. Additionally, atomic force microscopy (AFM) analysis was used to prove the successful coating preparation. The surface roughness (Ra) of SHPO‐Ber was 147.0 nm, while the Ra of MSNs was 104.0 nm. As shown in Figure 2e, the AFM images showed that the thickness of SHPO‐Ber (743.6 nm) was significantly higher than the MSNs surface (449.0 nm). Both of the results illustrated that the superhydrophobic coating was successfully reassembled on the MSNs‐modified surface.

### 2.2 Drug Loading and Release Profile of the SHPO‐Ber Coating

**FIGURE 3:**
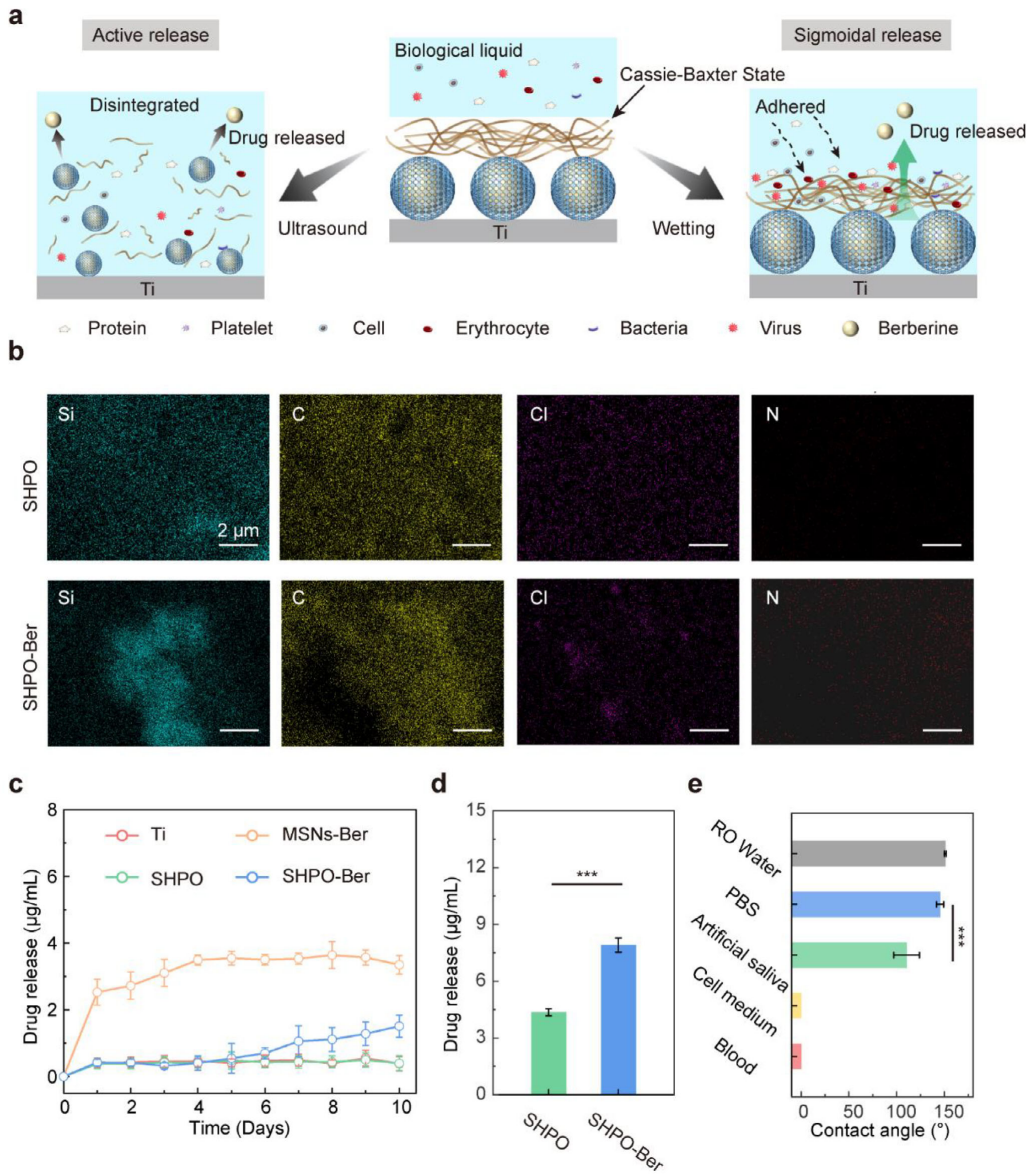
Drug loaded and released of SHPO‐Ber. (a) Schematic of the SHPO‐Ber drug release through the change of interfacial wettability and ultrasound. (b) SEM‐EDS analysis of the Ber loaded of the SHPO‐Ber abutments. (c) The Ber release curve of different abutment coatings. (d) The Ber release from the SHPO‐Ber by ultrasound. (e) The water contact angle of SHPO‐Ber after immersed in different liquids. (n = 3, analyzed using paired t test, ^***^ *p* < 0.001).

Usually, the superhydrophobic surface presents a Cassie–Baxter state to maintain the liquid‐repellent properties, and it could resist biological contamination and achieve anti‐fouling functionality [23]. However, with prolonged exposure to the biological fluids, the defects and protein accumulation on the superhydrophobic surface transformed to the Wenzel state [16]. The transformation of interfacial wettability altered drug release behavior, enabling the coating to function as a “drug reservoir” where Ber was reserved. The infiltration and wetting of superhydrophobic surfaces had become the switch that triggers the reservoir to release Ber. As shown in Figure 3a, Ber was released from the SHPO‐Ber coating with the protein adhesion and surface wetting after prolonged contact with biological fluids. Besides that, the “drug reservoir” can also be triggered actively under ultrasound stimulation, which could directly damage the structure of the superhydrophobic surface so that Ber could be rapidly released into the surrounding tissues.

To evaluate the Ber‐loading status on SHPO‐Ber coating, the SEM‐EDS was used to analyze. As shown in Figure 3b, it showed that the carbon element was evenly distributed, similar to the silicon element. Chlorine had a localized enrichment at the sites of nanoparticle accumulation, which revealed that the OTS successfully coated and reacted with the MSNs surface. Importantly, after Ber being loaded into SHPO, the nitrogen element was significantly improved (Table S2), and the nitrogen element fluorescence distribution was also increased.

To accurately simulate the drug release in the oral environment, the artificial saliva was chosen to test the drug release profile. As shown in Figure 3c, the MSNs‐Ber showed a typical burst release behavior at day 1, while the SHPO‐Ber only presented Ber release between day 6 and day 7. At the same time, the Ti and SHPO groups had no Ber release as expected. The Ber release curve was regulated by the wettability transition. During the first 6 days of soaking, no Ber release was observed. The Ber release only started with the superhydrophobic coating wetted, and maintained stably in the later stage, which made the drug release behavior become sigmoidal. Ber drug release concentration reached more than 1 µg/mL, which achieved a therapeutically effective level [24, 25]. The network structure formed by hydrophobic micelles via self‐assembly also provided possible channels for drug release, preventing the drug from being completely encapsulated. In addition, the active control of drug release through ultrasound stimulation. Ber released from the SHPO‐Ber coating was significantly increased compared with the SHPO group (Figure 3d and Figure S2). The reason for the drug release of SHPO might be the residual MSNs affecting the absorption and emission of UV. But in any case, the ultrasound stimulation was beneficial for Ber's release from both the coating and MSNs.

In order to specifically investigate the wettability changes of the coating, various liquids were used to study the interface regulation (Figure 3e). After one week of immersion in different liquids, blood and cell medium could induce complete surface wetting. However, RO water and PBS could not change the surface from the Cassie–Baxter to the Wenzel state, although PBS slightly decreased the contact angle due to its rich ionic composition. The SHPO‐Ber coating soaked in the artificial saliva, which showed that the water contact angle was 109.8°. The results explained that the existence of proteins and cells plays an important role in the interfacial wettability transition, while multiple ionic components would also have some effects [26, 27].

### 2.3 Antibacterial and Anti‐Inflammatory Function of the SHPO‐Ber Coating via Sigmoidal Drug Release

**FIGURE 4:**
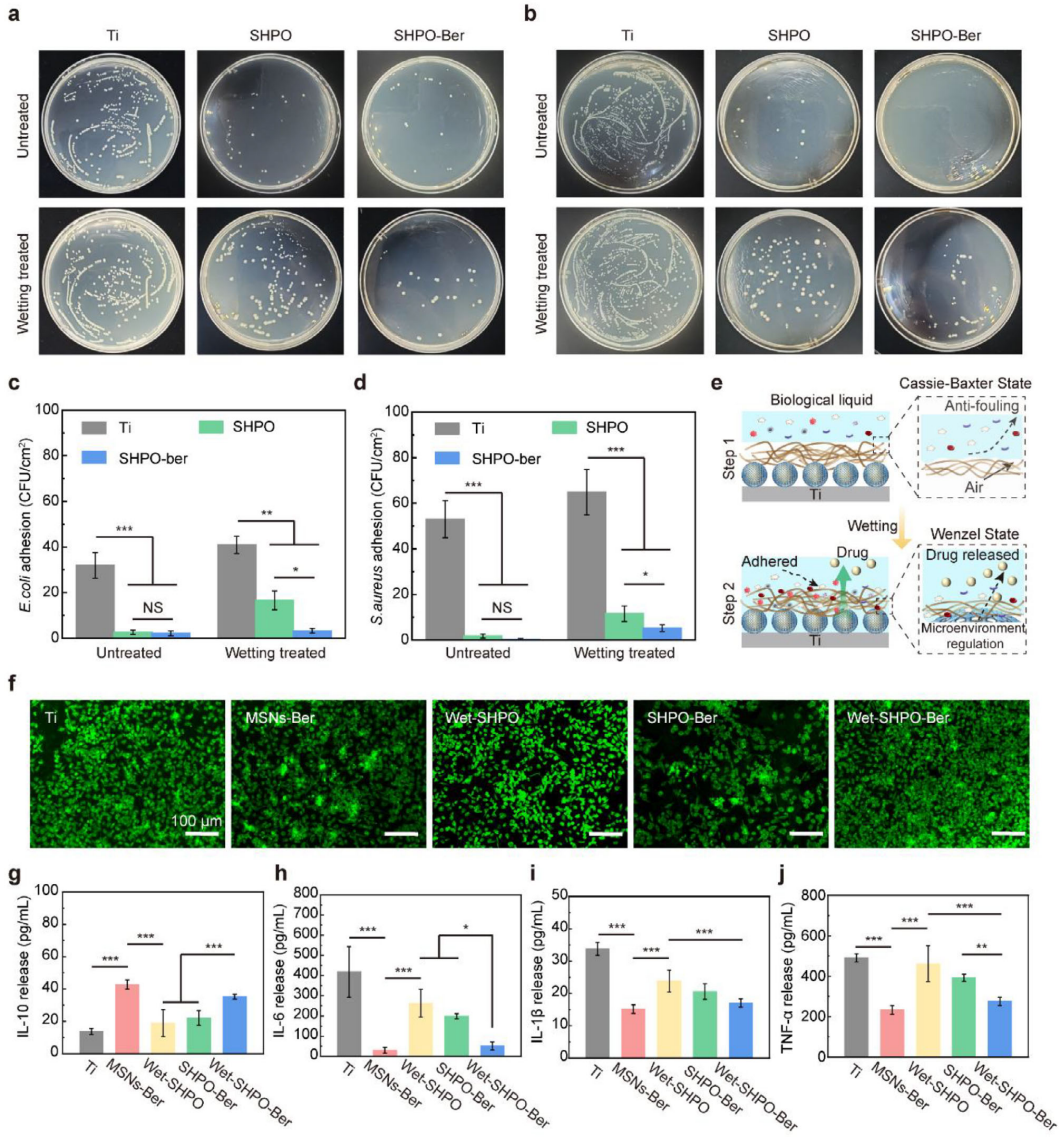
Dual‐phase functionalization of SHPO‐Ber. (a) Plate photographs for *E. coli* that adhered on different samples with and without wetting treated at 24 h. (b) Plate photographs for *S. aureus* that adhered on different samples with and without wetting treated at 24 h. (c) Number of *E. coli* that adhered on different samples with and without wetting treated at 24 h. (d) Number of *S. aureus* that adhered on different samples with and without wetting treated at 24 h. (e) Schematic of the SHPO‐Ber exerting the coating functions at different wetting states. (f) Fluorescence images of rhodamine 123 staining of RAW264.7 induced with the LPS cocultured with different samples for 1 day. Inflammatory factors (g) IL‐10; (h) IL‐6; (i) IL‐1β; (j) TNF‐α; release from the RAW264.7 induced with the LPS cocultured with different samples for 1 day. (n = 3, analyzed using one way ANOVA, ^*^ *p* < 0.05).

The sigmoidal drug release coatings prepared in the implant abutment could have dual‐phase functionality mediated by the transition from Cassie–Baxter to Wenzel wetting state. In this way, the SHPO‐Ber coating simultaneously achieves antifouling in the Cassie–Baxter state (group setting method shown in Table S3) and microenvironment regulation in the Wenzel state (group setting method shown in Table S4). To evaluate antibacterial performance, *Escherichia coli* (*E. coli*) and *Staphylococcus aureus* (*S. aureus*) were used as model bacteria (Figure 4a,b) to test whether the construction of the surface superhydrophobic coating has enabled the early realization of antibacterial adhesion ability. And coatings were pre‐soaked in artificial saliva for a week to obtain wetting samples (Wet‐SHPO, Wet‐SHPO‐Ber), which were used to test the antibacterial properties of the drug released from the coating, after the coating becomes wet. As shown in Figure 4c, for *E. coli*, the untreated group showed that the superhydrophobic surface had remarkable anti‐bacterial adhesion ability, reducing the *E. coli* adhesion amount by 12.3‐fold compared to controls, regardless of drug loading. Compared with the MSNs‐Ber group, SEM images showed that there were almost no bacteria adhering to the SHPO‐Ber surface, unlike the effect of antibacterial drugs (Figure S3). After the samples were wetting treated, the superhydrophobic surfaces retained significantly anti‐fouling performance. Especially, SHPO‐Ber reduced 12.6‐fold bacteria adhesion compared to bare Ti, which demonstrated Ber release in the Wenzel state had significantly antibacterial performance. Similarly, the untreated group showed that the superhydrophobic surface had remarkable anti‐*S. aureus* adhesion (30.3‐fold), when SHPO‐Ber also showed that the anti‐*S. aureus* in the Wenzel state (Figure 4d). Furthermore, when *Porphyromonas gingivalis* (*P.gingivalis*) was used to evaluate the antibacterial performance of SHPO‐Ber, the results aligned with those obtained from *E. coli* and *S. aureus*. It demonstrated that regardless of incubation time (24 or 72 h), both the superhydrophobicity of the SHPO surface and the drug release triggered upon wetting of the SHPO‐Ber surface contributed positively to the antibacterial efficacy of the coating (Figure S4).

Therefore, the sigmoidal drug‐release coating established by the superhydrophobic nanoparticles could have a dual‐phase function with the wettability transition. As shown in Figure 4e, the SHPO‐Ber coating on the implant abutment presented a Cassie–Baxter state at step 1, which could provide anti‐fouling ability when contacting with the biological fluid. When plenty of proteins and cells adhered onto the surface, the SHPO‐Ber coating presented the Wenzel state at step 2. In this state, Ber loaded in SHPO‐Ber could be released in a sustained manner and exerted the therapeutic effect.

Regarding the early‐stage effect of the drug after release, the most important thing was that Ber had anti‐inflammatory and antioxidant properties around dental implants. Therefore, the anti‐inflammatory functions of drug‐loaded and SHPO samples were tested by being cocultured with the LPS‐stimulated macrophages (RAW 264.7) [28]. As shown in Figure 4f, the morphology of LPS‐stimulated cells was featured by many extended tiny protrusions, although cell activity had no significant difference (Figure S5). Analysis of inflammatory factors secreted by cells revealed that Ber release from the coatings upregulated the anti‐inflammatory cytokine IL‐10 (Figure 4g). Meanwhile, the level of pro‐inflammatory factors IL‐1β, IL‐6, and TNF‐α was reduced in the presence of Ber‐releasing coatings (Figure 4h–j). Ti, Wet‐SHPO, and SHPO‐Ber had no obvious inflammatory regulatory effects. Simultaneously, the Wet‐SHPO‐Ber and MSNs‐Ber coating showed significant downregulation of pro‐inflammatory cytokines, which further explained that Ber had the anti‐inflammatory ability. Compared with the SHPO‐Ber, the pro‐inflammatory factors release of the Wet‐SHPO‐Ber group was significantly decreased, which also proved that the wetting state transition influenced the sequential nature of drug action.

Ber also had the antioxidant performance to regulate the microenvironment of the dental implantation site, which could clear the abnormal ROS to support the peripheral wound healing and bone integration [29, 30]. Through the DPPH and ABTS scavenging test (Figure S6), the coatings with Ber (MSNs‐Ber and Wet‐SHPO‐Ber) had significantly improved antioxidant capacity compared to bare Ti and SHPO‐Ber coatings, demonstrating that Ber could effectively scavenge excess ROS around the implant.

### 2.4 Hemocompatibility and Biocompatibility Evaluations

**FIGURE 5:**
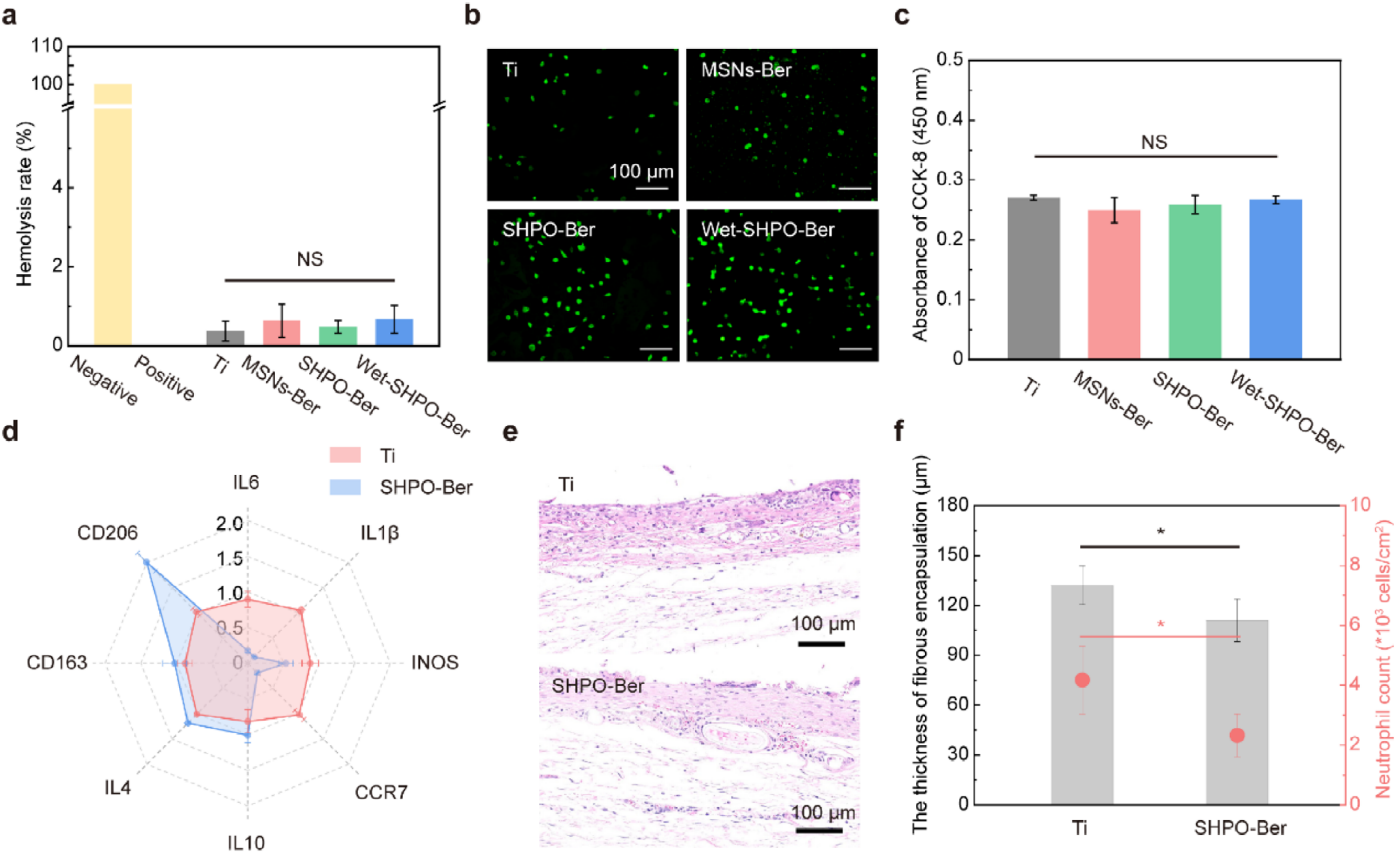
Hemocompatibility and biocompatibility evaluations. (a) Hemolysis ratio analysis of various samples and their pictures. (b) Fluorescence images of rhodamine 123 staining of L929 cells cultured with different samples for 1 day. (c) CCK‐8 of the viability of L929 cells cocultured with different samples at day 1. (d) Radar chart analysis of the anti‐inflammatory behaviors by PCR after RAW264.7 cocultured with different samples. (e) Images of HE staining with different samples implanted subcutaneously in SD rats for 7 days. (f) Capsule thickness and Neutrophil count on the samples after subcutaneous implantation into SD rats. (n  = 3, ^**^ *p* < 0.01, ^***^ *p* < 0.001).

As a part of dental implants, the abutment and its coatings must provide good biocompatibility to avoid adverse reactions and post‐implantation complications, which is a critical prerequisite for clinical application. As shown in Figure 5a, the hemolysis rates of bare Ti and SHPO‐Ber coatings were below 1.0% and had no statistically significant difference, indicating their minimal risk of hemolysis upon contact with blood. Then, the L929 cells were used to evaluate cell activity and compatibility, because fibroblasts were the most important cells in the gingival connective tissue and can provide strength and structural support for soft tissues [31]. As shown in Figure 5b, cell morphology remained unaffected after coculture with various samples, and their cell activity also had no significant difference (Figure 5c). These results demonstrated that both the superhydrophobic coating and MSNs had good biocompatibility. Through PCR analysis, the inflammation‐related factors, the Ti and SHPO‐Ber cocultured with RAW264.7. The pro‐inflammatory related factors (IL‐6, IL‐1β, CCR7, and iNOS) were significantly inhibitory release when regulated by SHPO‐Ber. And anti‐inflammatory related factors expression (IL‐10, IL‐6, CD163, and CD206) were significantly increased, especially CD206, which showed that the SHPO‐Ber could be conducive to the polarization of macrophages into M2‐type (Figure 5d and Figure S7) [32, 33].

Furthermore, the subcutaneous implantation experiment was used to evaluate the immune response and histocompatibility of implant materials. After the samples were implanted under the rat backs for 7 days, the tissue was stained by HE as shown in Figure 5e. Through analyzing the thickness of fibrous encapsulation, the SHPO‐Ber showed that the thickness of proliferative tissue was about 121.9 µm, which was thinner than Ti (131.1 µm). Then, the neutrophil of the tissue contacted with SHPO‐Ber was significantly less than the tissue contacted with Ti (Figure 5f). This indicated that the SHPO could reduce endometrial hyperplasia and regulate the local inflammation [34, 35].

### 2.5 Implant Model Establishment and In Vivo Evaluations

**FIGURE 6:**
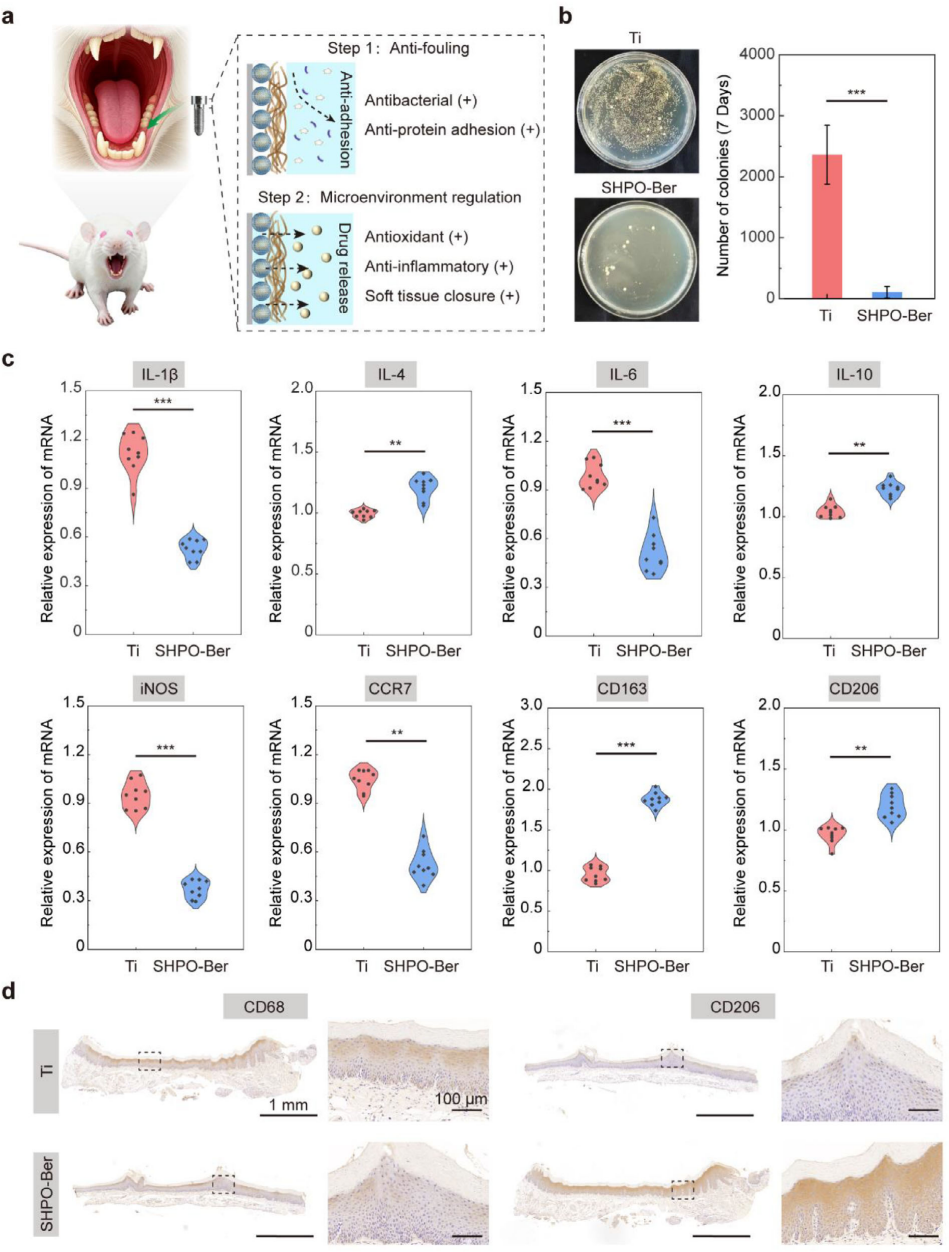
Implant model establishment and evaluations in vivo. (a) Schematic of the abutments with SHPO‐Ber implanted in the rats’ mouths. (b) The antibacterial behavior of SHPO‐Ber abutments after being implanted for a week. (n = 5, analyzed using paired t test, ^***^ *p* < 0.001). (c) Analysis of the anti‐inflammatory behaviors by PCR after SHPO‐Ber abutments were implanted for two weeks. (d) Immunohistochemistry staining (CD68/CD206) images of tissue around Ti and SHPO‐Ber abutments for two weeks. (n = 6, analyzed using paired t test, ^***^ *p* < 0.001).

The Ti nail implant was manufactured using laser cutting, and the basic parameters are shown in Figure S8. The abutment of the Ti implant was coated with SHPO‐Ber, and it was implanted into the oral cavity of rats to analyze the dual‐phase functionality of the SHPO‐Ber coating, including the anti‐fouling in step 1 and the microenvironment regulation in step 2 (Figure 6a). After 7 days of implantation, bacterial adhesion on the implants was measured by the spread plate method. As shown in Figure 6b, the number of colonies adhering to the Ti was 22.5 times higher than that on the SHPO‐Ber coating, which demonstrated that the SHPO‐Ber surface had significantly anti‐fouling ability than pure Ti. It was mainly due to the anti‐fouling performance of the superhydrophobic coating in the early stage, which was consistent with the results of in vitro experiments [36].

After the implant was placed for two weeks, the effect of the implant on the surrounding tissues was also analyzed by PCR to characterize the regulation of the tissue microenvironment in the later stage. Specifically, pro‐inflammatory indicators such as IL‐6 and TNF‐α, and anti‐inflammatory indicators such as IL‐10 and CD163, were chosen to analyze the inflammatory regulation of the organizational microenvironment by the SHPO‐Ber coating. As shown in Figure 6c, the rats implanted with SHPO‐Ber showed that the expression of anti‐inflammatory‐related genes was markedly elevated, while the expression of pro‐inflammatory‐related genes was significantly decreased. This result was consistent with the established pharmacological properties of Ber. Ber exhibited anti‐inflammatory effects via inhibiting the activation of related inflammatory factors such as iNOS, IL‐6, and TNF‐α, and it also could reduce the stimulating effect of LPS on macrophage inflammation [37]. The tissue around the samples was also immunohistochemistry staining with CD68/CD206 (Figure 6d), which showed that the SHPO‐Ber exhibited a higher proportion of M2 compared to the control group. All the results indicated that SHPO‐ber exhibited anti‐inflammatory properties upon implantation at a late stage.

The tissue around the implants was stained by HE (Figure S9). The results showed that the tissue around the SHPO‐Ber implant had a low inflammatory response, as it had fewer neutrophils. Simultaneously, the Masson staining also illustrated that the SHPO‐Ber coating promoted the formation of stable and mature fibrous tissue. To evaluate systemic biosafety, major organs (heart, liver, spleen, lung, and kidney) of rats treated with the dental implant placement were observed following HE staining for histological assessment. As shown in Figure S10, the SHPO‐Ber coating caused no damage to these organs, indicating that the coating had good biosafety and biocompatibility.

### 2.6 Evaluations of Soft Tissue Closure in the Rat Maxillary Implant Model

**FIGURE 7:**
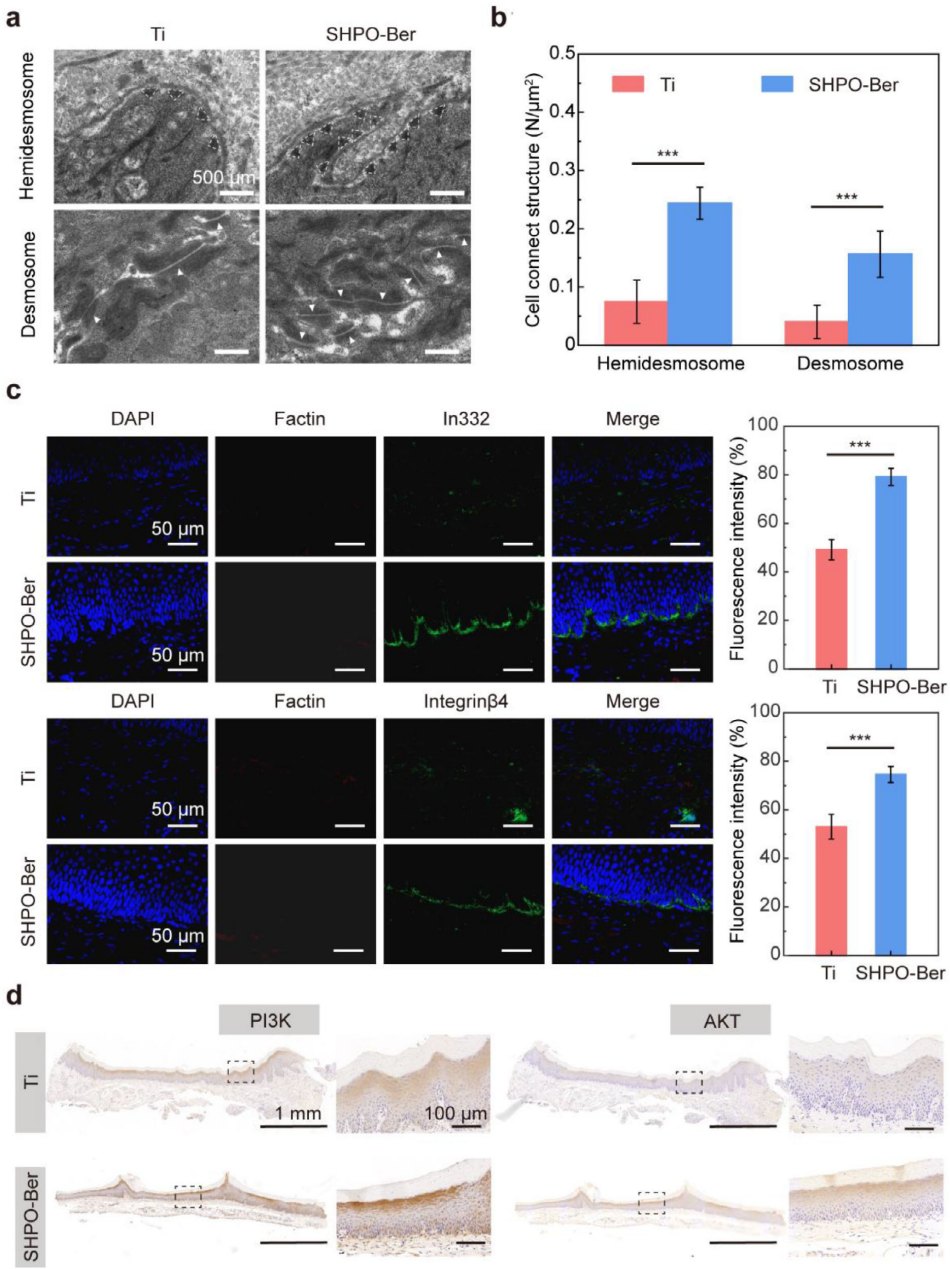
Evaluations of soft tissue closure in a rat maxillary implant model. (a) TEM images of the hemidesmosome and desmosome treated with Ti and SHPO‐Ber abutments. (b) Hemidesmosome and desmosome statistics treated with Ti and SHPO‐Ber abutments. (c) Immunofluorescence staining images and quantified fluorescence intensity of laminin 5 and integrin β4 in tissue around Ti and SHPO‐Ber abutments. (n = 5, analyzed using paired t test, ^***^ *p* < 0.001).

After implantation, the most important function of the implant abutment is to promote soft tissue closure [38]. At the 2 and 4 weeks post‐implantation, the soft tissue around the abutment with the SHPO‐Ber coating exhibited superior healing, whereas the soft tissue around pure Ti had observable inflammatory reactions such as edema and fester (Figure S11). To further evaluate the tissue‐implant interface, the hemidesmosome and the desmosome of tissues were observed and counted under the TEM, which plays a crucial role between the implant and the soft tissue (Figure 7a). As shown in Figure 7b, the tissue around the SHPO‐Ber had significantly increased hemidesmosome (3.92‐fold) and desmosome (3.27‐fold), which illustrated that the SHPO‐Ber coating significantly promotes the epithelial cell interaction and the attachment of epithelial cells to the underlying connective tissue. By analyzing the epithelial attachment length and collagen volume fraction (CVF) in peri‑implant tissues confirmed that the SHPO‑Ber coating significantly enhanced both epithelial attachment extension and tissue maturation. These results demonstrate that the SHPO‑Ber coating actively supports tissue regeneration and structural maturation at the implant–tissue interface (Figure S12).

The expression of integrin β4 and laminin 332 in the tissues was further analyzed by fluorescence staining. As shown in Figure 7c, it could be observed that there was only a little expression of integrin β4 and laminin 332 around the Ti implant. In contrast, a complete band distribution of these two proteins at the tissue interface was observed when SHPO‐Ber was coated on the implant. Quantitative analysis of fluorescence intensity revealed that the expression levels of integrin β4 and laminin 332 in the SHPO‐Ber group were approximately 74.6% and 79.1%, respectively, which were significantly enhanced compared with the Ti group (about 53.1% and 49.1%, respectively). The high expression of integrin β4 and laminin 332 in the SHPO‐Ber group is conducive to strongly coupling the keratin cytoskeleton and the extracellular matrix, thus maintaining the polarity and stratified structure of epithelial cells, so that the SHPO‐Ber coating could promote the closure of soft tissues around the implant [39, 40, 41]. To explore the mechanism by which this coating promotes soft tissue closure, we performed immunohistochemical staining on the tissues. As shown in Figure 7d, the SHPO‐Ber group exhibited markedly higher expression of PI3K/AKT compared to the control group. Therefore, the enhanced soft tissue healing can be attributed to berberine‑mediated activation of the PI3K/AKT signaling pathway, which promoted cell survival, migration, and tissue repair.

## 3 Conclusion

In this study, the sigmoidal and sequential drug release pattern was proposed for dental implant abutment to avoid peri‐implantitis and promote soft tissue closure. The SHPO‐Ber coating integrated the anti‐fouling characteristics of superhydrophobic surfaces with controlled drug release by the property of the wettability state change at the superhydrophobic interface, which was different from the previous drug release patterns. The superiority of this design manifested in three critical aspects. First, the superhydrophobic shell had universal anti‐fouling performance for all components of biological liquids. It can react with the drug carriers to form a stable coating via reassembly, and it has lower biological toxicity due to its fluorine‐free composition. Second, Ber could be easily loaded into the common drug carrier (MSNs) to ensure drug stability within the coating. Ber, as a botanical product, has been reported to be used in antibacterial and anti‐inflammatory areas, which promoted the proliferation and migration of vascular endothelial cells that are beneficial for soft tissue closure [26, 42]. Third, the system effectively integrates the anti‐fouling function of the superhydrophobic surfaces with the functionality of Ber, exerting a synergistic effect in biomedicine. For example, when massive bacterial adhesion onto the superhydrophobic shell alters interface wettability, Ber release is triggered and sustained to enhance the antibacterial property. It also presented the unique functional mechanism that combined microenvironment regulation and anti‐fouling into a single drug release platform. Meanwhile, the SHPO‐Ber coating simultaneously exhibits two modes of intelligent drug release. For active on‐demand release, the SHPO‐Ber could quickly release the drug by destroying the coating through ultrasonic stimulation. It illustrated that active Ber release can be controlled artificially to suit the appropriate stage of treatment, and the Ber release rate is increased when ultrasound breaks the physical binding between Ber and MSNs. For condition‐responsive release, the superhydrophobic coatings exhibited distinct wetting behaviors in biological liquids of different components. High concentrations of biological components in liquids would accelerate the wettability transition. Usually, high concentrations of biological components meant the disease around the implant was more serious, which could enhance the rate of drug release with the change in interfacial wettability.

As a promising strategy, the preparation method and drug selection of the superhydrophobic coating were not unique. Through various micro‐nano processing technologies (lithography, spinning, microneedles, et al.) and the plenty of preparation schemes of superhydrophobic coatings (dip coating, spray coating, CVD, et al.), the superhydrophobic drug delivery coating can be designed to address different clinical needs. For example, this coating for the wound dressings can provide early‐stage antibacterial protection and late‐stage microenvironment regulation; on the vascular stent, this coating can offer early anticoagulation and later promote reendothelialization of the stent surface to prevent restenosis; in tumour therapy, this coating can prolong the circulation time of nanocarriers in the body. Therefore, the drug release mode based on wettability transition at the superhydrophobic interface provides a universal strategy for various clinical health issues.

In conclusion, the SHPO‐Ber coating used in the implant abutment based on interface wettability regulation, achieved early antibacterial and antifouling properties at the superhydrophobic state. Simultaneously, it demonstrated that it can regulate the microenvironment and promote the soft tissues closure after coating wetting. The sigmoidal drug release mode offered a novel development direction to deal with the challenges of low drug utilization and non‐intelligent clinical treatment.

## 4 Experimental Section

### 4.1 Materials

Mesoporous silica (Outer diameter 450–550 nm, aperture 2–4 nm), octadecyltrichlorosilane (OTS, ≥ 90%), and lipopolysaccharide (LPS from *E. coli* 055: B5) were all purchased from Shanghai Aladdin Biochemical Technology Co., Ltd (China). FITC‐BSA was purchased from Beijing Bersee Technology Co. (Beijing, China). Artificial saliva (Greenwood) was purchased from Leagene Bio Co. (Beijing, China). IL‐1β, IL‐6, IL‐10, and TNF‐α ELISA kits were purchased from Beijing Solarbio Science & Technology Co. (Beijing, China) and Wuhan Jinyinmei Co. (Wuhan, China), respectively. Rhodamine 123, was purchased from Sigma‐Aldrich (USA). Cell counting kit‐8 (CCK‐8) was purchased from Shanghai Beyotime Biotechnology (Shanghai, China). 1,1‐diphenyl‐2‐trinitrophenylhydrazine (DPPH) and Berberine chloride (Ber, 98%) were purchased from Macklin (China). *Escherichia coli* (*E. coli*, ATCC‐700926) and *Staphylococcus aureus* (*S. aureus*, ATCC‐6538) were provided by Southwest Medical University. Materials for cell culture, including Trypsin (0.25%), Penicillin, Streptomycin, DMEM (Dulbecco's Modified Eagle Medium) Basic medium, DMEM High Glucose medium, Fetal bovine serum (FBS), Luria–Bertani (LB) medium, were all purchased from Wuhan Saiweier Biotechnology Co., Ltd (Wuhan, China). Hexane, ethanol, and acetone were purchased from Chengdu Kelong Co. (Chengdu, China).

### 4.2 Preparation of the SHPO‐Ber Coating

The berberine chloride (Ber, 20 mg) and mesoporous silica nanoparticles (MSNs, 100 mg) were mixed in the RO water (20 mL), and treated by ultrasound for 2 h to load Ber into MSNs. Then, the residual Ber in solution was washed with RO water for 3 times at 3000 rpm centrifugation. The berberine chloride‐loaded mesoporous silica nanoparticles (MSNs‐Ber) were obtained after being dried at 60°C for 12 h.

The hydrophobic micelles had been reported in previous work [20]. Briefly, 20 µL Ber solution (20 mg/mL in RO water) was added to 1 mL OTS in 2 mL EP tube. The EP tube was sequentially subjected to three cycles of vortex followed by ultrasonication. After 2 h without seal, the mixture solution was transferred to 10 mL hexane. Through shaking, the hydrophobic micelles were obtained for use, and the mixture solution should be freshly prepared and used promptly.

The superhydrophobic drug‐loaded coating (SHPO‐Ber) was prepared by two‐step dipping. Before preparation, the Ti sheet and Ti foil were cleaned three times with ethanol, acetone, and RO water by ultrasound for 10 min. For the first step, Ti was dipping into the MSNs‐Ber solution (20 mg/mL dispersed with water) for 10 s to deposit MSNs‐Ber on the Ti surface, and it was heated and solidified at 60°C for 12 h. For the second step, Ti with MSNs‐Ber was dipping into the shaken hydrophobic micelle solution for 1 min, and it was also heated and solidified at 60°C for 12 h to get the SHPO‐Ber coating. For other groups, SHPO samples were prepared similarly to SHPO‐Ber, except for using MSNs without Ber loading.

### 4.3 Characterization of Coating Performance and Structure

Water contact angle was measured by the CA measuring instrument (OCA50AF, DataPhysics Instruments, Filderstadt, Germany). 5 µL of RO water was added on sample surfaces, followed by calculation with the Young's modulus. All water contact angles were measured at least 5 times.

Morphology of surface coatings and nanoparticles was observed by scanning electron microscopy (SEM) (GeminiSEM300, Zeiss, Oberkochen, Germany). Surface element compositions were also analyzed by Energy Dispersive Spectrometer (EDS).

Anti‐protein adhesion evaluation was conducted based on the model protein FITC‐BSA. FITC‐BSA was dissolved in PBS at 2.5 mm to prepare the work solution. 1 mL of work solution was added to cover the samples in 24‐well plates for 1 day at 37°C. After incubation, sample surfaces were gently washed for 3 times with PBS followed by observation using a fluorescence microscope (EVOS M5000, Thermo Fisher Scientific). The fluorescence area was analyzed with Image J.

For the anti‐fouling stability test, samples were immersed in RO water, PBS, porcine blood, FITC‐BSA solution, and cell culture medium for one week. After the liquid on the surface was gently wiped off, the contact angle on the surface of the samples was tested.

### 4.4 Drug Release Test

Samples (0.5^*^0.5 cm) were placed into a 24‐well plate with 1 mL artificial saliva for incubation at 37°C. At 1, 2, 3, 4, 5, 6, 7, 8, 9, and 10 days, 100 µL of soaking solution was taken to test the absorbance at 345 nm by the microplate reader, and the same volume of corresponding fresh artificial saliva was replenished.

Drug release of the sample was also tested by active ultrasound (40 kHz, 100 W). Briefly, samples were completely immersed in 1 ​mL artificial saliva, and it was treated by ultrasonication for 1 min to accelerate drug release. 100 ​µL of soaking solution was taken to test the absorbance at 345 nm by the microplate reader.

### 4.5 Antibacterial Properties

To analyze the antibacterial ability of the coating in two stages, some SHPO‐Ber samples were immersed in fresh artificial saliva for 7 days to make the coating wetted and the drug of samples exposed (Wet‐SHPO‐Ber).

Classic Gram‐negative bacteria (*E. coli*) and Gram‐positive bacteria (*S. aureus*) were as model bacteria. Samples (1^*^1 cm) were placed into a 24‐well plate and sterilized by UV. *E. coli* and *S. aureus* were grown in 5 mL LB for approximately 6 h at 37°C. The concentration of bacteria was diluted to 10^8^ CFU/mL, and it was diluted by PBS to 5^*^10^6^ CFU/mL and divided 1 mL to a 24‐well plate to immerse the samples. After 24 h culture at 37°C, samples were taken out and washed gently with PBS. Then, samples were transferred to 2 mL PBS to detach adhering bacteria by vortexing for 1 min. The bacterial solutions were diluted 100 times, and 40 µL of bacteria dilution solution was applied to the coating plate and cultured at 37°C for 24 h. The plate was photographed to calculate the bacterial colony number using Image J. At the same time, the bacteria adhered to the samples were fixed by 4% paraformaldehyde for the whole night, followed by gradient dehydration by 50%, 75%, 90%, 95% ethyl alcohol and anhydrous ethanol for SEM observation.

### 4.6 Hemolysis Test

Human whole blood was collected from healthy donors with either sodium citrate or EDTA at a volume ratio of 1:9 (anticoagulant to blood) for anticoagulation. The fresh blood was then diluted with normal saline to a concentration of 2%. Samples were placed in EP tubes and submerged in the 2% blood solution. All test groups (1^*^1 cm), including normal saline (serving as a negative control) and reverse‐osmosis water (as a positive control), were incubated in a water bath at 37°C for 2 h. After incubation, samples were centrifuged at 5000 rpm for 5 min. The absorbance of the supernatant was measured at 540 nm using a microplate reader, and the hemolysis percentage was determined according to the formula:

(1) $$\mathrm{Hemolysis}\;\mathrm{rate}\;\;\left(\%\right)=\frac{A-B}{C-B}\;\times 100\%$$

where A is the absorbance of the experimental group, B is the absorbance of the negative control group, and C is the absorbance of the positive control group.

### 4.7 Cell Activity and Anti‐Inflammatory Properties Test

Macrophage (RAW 264.7) and fibroblast (L929) were used to evaluate the biocompatibility properties. Samples (1^*^1 cm) were prepared in a 24‐well plate and sterilized under UV light. Subsequently, 1 mL of L929 suspension, at a density of 1 × 10^4^ cells/mL in high‐glucose DMEM supplemented with 10% FBS, was seeded onto each sample for coculture. After 24 h of culture, cell proliferation was assessed using a CCK‐8 assay (CCK‐8: Cell culture medium = 1:9(v/v)). After 3 h incubation with the CCK‐8 reagent, the absorbance of the supernatant was measured at 450 nm using a microplate reader. In parallel, another set of samples was fixed overnight in 2.5% glutaraldehyde. These fixed cells were then stained with rhodamine 123 and imaged under a fluorescence microscope (EVOS M5000, ThermoFisher, USA). For RAW 264.7, the different procedure was the change of the original culture medium to LPS medium (Lipopolysaccharides, 50 ng/mL in DMEM cell culture medium) to induce inflammatory responses, and the cell density was 1 × 10^5^ cells/mL.

To evaluate the anti‐inflammatory properties of coatings, the RAW 264.7 culture medium was collected and analyzed using IL‐1β, IL‐6, IL‐10, and TNF‐α ELISA kits.

Following a 3‐day coculture with the biomaterial, total RNA was extracted from macrophages and reverse‐transcribed into complementary DNA using reverse transcriptase and primers. The expression of specific genes was then quantified by real‐time quantitative polymerase chain reaction (qRT‐PCR). To assess macrophage polarization, primers for the M1 markers (iNOS and CCR‐7) and the M2 markers (CD206 and CD163) were used alongside primers for key cytokines (IL‐1β, IL‐6, IL‐4, IL‐10). The housekeeping gene glyceraldehyde‐3‐phosphate dehydrogenase (GAPDH) served as an internal control. Fluorescence was monitored in real‐time during amplification, and the relative expression levels of the target genes were normalized to GAPDH and calculated using the 2^(‐ΔΔCT) method.

### 4.8 ROS Scavenging Ability Test

The ROS scavenging efficiency of the samples was evaluated using DPPH and ABTS. Samples (1^*^1 cm) were placed in the 24‐well plate, and DPPH (0.1 mmol/L in PBS) was added to immerse them for 30 min. The absorbance at 517 nm was measured using a UV, and the DPPH scavenging ability was calculated as Equation (2).

(2) $$DPPH\;scavenging\;ability\;\;\left(\%\right)=\frac{A_{DPPH}-A_{Sample}}{A_{DPPH}}\;\times 100\%$$

where *A_DPPH_* and *A_Sample_* represented the absorbance of the DPPH solution without and with samples. And ABTS was tested by an ELISA kit.

### 4.9 Establishment of Animal Models and In Vivo Treatment

All procedures were performed in accordance with the Animal Protection Agreement of the China Animal Protection Association and Southwest Medical University, and all ethical guidelines for experimental animals were followed (No. 20220316‐003). SD rats (male, 220 g) were purchased by Dossy Co., LTD, Chengdu. After adapting to the living environment, SD rats were narcotized with Zoletil (30–40 mg/kg). First, a ridge incision was made, followed by the elevation of a full‐thickness mucoperiosteal flap on the mid‐buccal aspect of the left first molar and the crest of the alveolar ridge to expose the underlying bone. An osteotomy site was then prepared directly at the ridge crest, parallel to the long axis of the first molar, using a low‐speed handpiece (Lone Star Dental, Arlington, Texas) and a guided drill. The drill bit was marked to maintain a consistent preparation depth at each stage. After confirming the integrity of the nasal floor, a 1.5‐mm diameter screwdriver (Small Parts, Miami Lakes, Fla) was used to place the implant into the most coronal portion of the osteotomy site. Following implant evaluation, the flap was sutured if indicated. Sutures were removed one week postoperatively [43].

### 4.10 Anti‐bacterial Adhesion Performance Test in the Early Stage of Implantation

To evaluate the surface antibacterial performance in the early stage of implantation, implants were taken out to analyze the bacterial adhesion on the surface of the implant at day 7 (n = 5). Specifically, implants were taken out and gently rinsed with PBS. And they were put into 1 mL PBS to make the bacterial dispersion by vortex oscillation (1500 rpm, 5 min). After dilution for 1000 cycles, 50 µL of bacteria dilution solution was coated on the plate and cultured at 37°C for 24 h. The plate was also taken the photo to calculate the bacterial colony number by Image J.

### 4.11 In Vivo Inflammatory Regulatory Performance Test

The tissues around the implant were collected after 14 days of implantation, and the regulatory ability of the implant on the surrounding inflammatory response was tested by PCR (n = 6). The tissue surrounding the implants was dissected into fragments smaller than 0.5 cm^3^ and immediately placed in TRIzol reagent for RNA isolation. Tissue lysis was performed by grinding in liquid nitrogen or using electric homogenization. Homogenization was carried out thoroughly at a ratio of 100 mg tissue per 1 mL TRIzol. Following chloroform phase separation, the aqueous phase was collected, and RNA was precipitated with isopropyl alcohol, washed with 75% ethanol, and finally dissolved in RNase‐free water. Genomic DNA contamination was eliminated through DNase I digestion. The purified total RNA was reverse‐transcribed into complementary DNA (cDNA) using reverse transcriptase and oligo (dT) or random primers. Gene‐specific primers were designed for target inflammatory genes (including pro‐inflammatory factors TNF‐α and IL‐6, anti‐inflammatory factors IL‐10, et.), with GAPDH or β‐actin serving as the internal reference. Quantitative PCR was performed on a real‐time PCR system, and relative gene expression levels were calculated using the 2^(‐ΔΔCt) method.

### 4.12 In Vivo Evaluation of Soft Tissue Closure

To evaluate the ability of the samples to promote the closure of soft tissues around the implants (n = 5), the maxillary bone mass containing the implant was harvested at day 14 and fixed in 4% paraformaldehyde at 4°C for 24 h, followed by overnight immersion in 2.5% glutaraldehyde (in 0.1 M PBS) at 4 °C. After PBS wash for three times, samples were post‐fixed in 1% osmium tetroxide (OsO_4_) for 2 h. Gradient dehydration was then performed by immersing samples in a series of ethanol solutions (50% to 100%), with 15 min per concentration, and two additional changes of 100% ethanol to ensure complete dehydration. Subsequently, samples were transferred to a transitional solution of acetone and epoxy resin (1:1) for 1 h, followed by infiltration in pure epoxy resin overnight. After polymerization of the resin blocks, ultrathin sections (approximately 80 nm) were cut using an ultramicrotome. The sections were stained with uranyl acetate for 30 min and lead citrate for 5 min. Finally, the hemidesmosome and desmosome were observed under a transmission electron microscope (TEM) at an acceleration voltage of 80 kV.

### 4.13 Immunofluorescence Staining of Soft Tissues

The peri‐implant tissue was harvested and sectioned at day 14. The sections were permeabilized with 0.3% Triton X‐100 and blocked with 10% serum. Subsequently, they were incubated overnight at 4 °C with the following primary antibodies: integrin β4 (1:300; Affinity, USA) and laminin 332 (1:300; Bioss, China). After incubation, the corresponding secondary antibodies were applied to facilitate the specific binding. Cell nuclei were counterstained with DAPI for visualization. Immunofluorescence images were acquired using a confocal laser scanning microscope, and fluorescence intensity was quantified with Image J software.

### 4.14 In Vivo Analysis of Tissue Toxicity and Inflammation

Upon completion of the 2‐week implantation period, the peri‐implant tissue and major organs (heart, liver, spleen, lung, and kidney) were harvested and fixed in 4% paraformaldehyde. All specimens were subsequently embedded in paraffin, sectioned, and subjected to hematoxylin and eosin (HE) staining for histological examination. Additionally, Masson's trichrome staining was performed specifically on the peri‐implant tissue sections. All stained sections were examined under a light microscope.

### 4.15 Subcutaneous Implantation Experiment

SD rats (male, 220 g) were purchased from Dossy Co., Ltd., Chengdu. After adapting to the living environment, samples were implanted under the skin of a rat back (n = 3). At day 7, samples were collected and fixed with 4% paraformaldehyde. Then, samples were embedded in paraffin, followed by tissue sectioning, and then observed by a microscope after HE staining.

### 4.16 Statistical Analysis

All experiments were performed at least three times, and the results are expressed as mean ± standard deviation (SD). One‐way analysis of variance (ANOVA) was used to analyze the experimental results, and the statistical difference between the two groups was considered significant when *p* < 0.05.

## Author Contributions

L.F., B.L. X.H., and D.Z. conceived the project and designed the experiments. Z.W., X.H., L.S., M.L., and J.W. provided assistance with designing the experiments. Z.W., X.H., L.S., carried out the fabrication, experiments, and characterization. B.L., Z.W., D.Z., G.W., and L.F. worked on the analysis of experimental results. X.H., B.L., and L.S. provided assistance with schematic drawing. B.L., L.F., L.S., and D.Z. wrote and revised the manuscript. All authors discussed the results and commented on the manuscript.

## Conflicts of Interest

The authors declare no conflicts of interest.

## Supporting information




**Supporting File**: advs75351‐sup‐0001‐SuppMat.docx.

## Data Availability

The data that support the findings of this study are available from the corresponding author upon reasonable request.
