# Supplementary material for: Interface Wettability Transition‐Driven Drug Release and Dual‐Phase Functionalization in Implant Abutment
Source: Adv Sci (Weinh). 2026 Apr 16;13(39):e75351. doi: 10.1002/advs.75351 (PMC13335656; doi:10.1002/advs.75351)
Supplement: Supplementary file 1 — Supporting File: advs75351‐sup‐0001‐SuppMat.docx. [file ADVS-13-e75351-s001.docx]

**Supporting Information**

**Interface wettability transition-driven drug release and dual-phase functionalization in implant abutment**

*Zhongchao Wang^145#^, Liang Shi^245#^, MingXia Li^345#^, Xiao Han^6^*, Jinghan Wang^245^, Guangping Wang^457^,* *Dan Zou^8^*, Bingyang Lu^6^*, Liyuan Fan^245^**

^1^ Department of periodontics &oral mucosal diseases, The Affiliated Stomatological Hospital, Southwest Medical University, Luzhou, Sichuan, China.

^2^ Department of Prosthodontics, The Affiliated Stomatological Hospital, Southwest Medical University, Luzhou, Sichuan, China.

^3^ Department of Oral Radiology, The Affiliated Stomatological Hospital, Southwest Medical University, Luzhou, Sichuan, China.

^4^ Luzhou Key Laboratory of Oral & Maxillofacial Reconstruction and Regeneration, Luzhou, Sichuan, China.

^5^ Institute of Stomatology, Southwest Medical University, Luzhou, Sichuan, China.

^6^ Institute of Fundamental and Frontier Sciences, University of Electronic Science and Technology of China, Chengdu 611731, P. R. China

^7^ Department of Orthodontics, The Affiliated Stomatological Hospital, Southwest Medical University, Luzhou, Sichuan, China.

^8^ School of Comprehensive Health Management, Xihua University, No. 9999, Hongguang Avenue, PiDu District, Chengdu, Sichuan Province, 610039, P.R.China

**Author information**

# Zhongchao Wang, Liang Shi and MingXia Li contributed equally to the article.

**Correspondence***

Liyuan Fan, Department of Prosthodontics, The Affiliated Stomatological Hospital, Southwest Medical University, Luzhou, Sichuan, China.

Email: [fly56@swmu.edu.cn](mailto:fly56@swmu.edu.cn)

Bingyang Lu, Institute of Fundamental and Frontier Sciences, University of Electronic Science and Technology of China, Chengdu 611731, P. R. China

Email: [bingyanglu9771@gmail.com](mailto:bingyanglu9771@gmail.com)

Dan Zou, School of Comprehensive Health Management, Xihua University, No. 9999, Hongguang Avenue, PiDu District, Chengdu, Sichuan Province, 610039, P.R.China

Email: [zoudan@xhu.edu.cn](mailto:zoudan@xhu.edu.cn)

Xiao Han, Institute of Fundamental and Frontier Sciences, University of Electronic Science and Technology of China, Chengdu 611731, P. R. China

Email: [xiaohan_xh@163.com](mailto:xiaohan_xh@163.com)


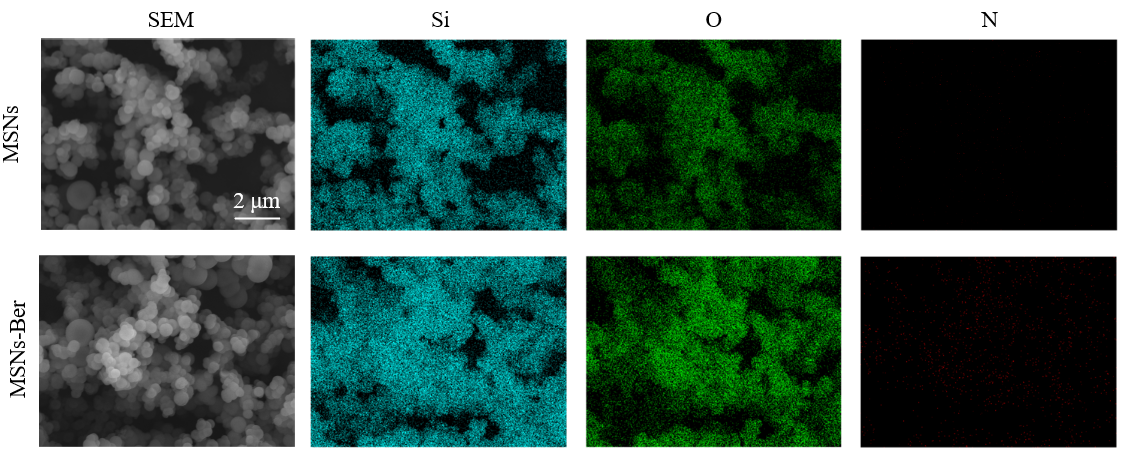


**Fig. S1** SEM-EDS analysis of the Ber loaded of the MSNs.


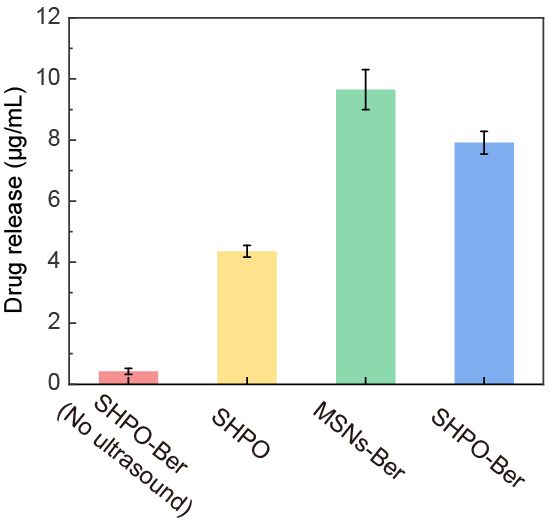


**Fig. S2** The Ber release from the various samples by ultrasound, and compared with the SHPO-Ber without ultrasound


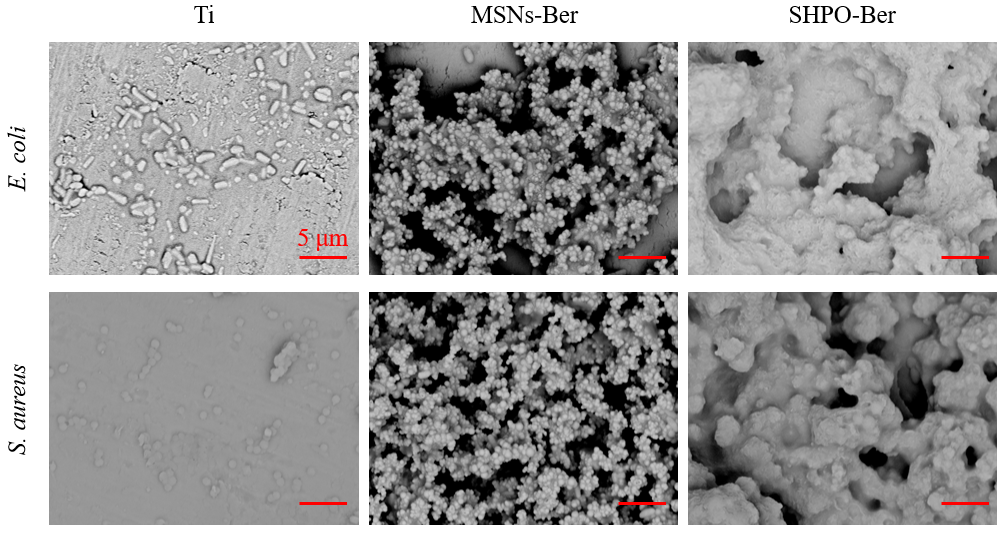


**Fig. S3** SEM images of the *E. coli* and *S. aureus* behaviour that adhered on different samples without wetting treated at 24 h.

**
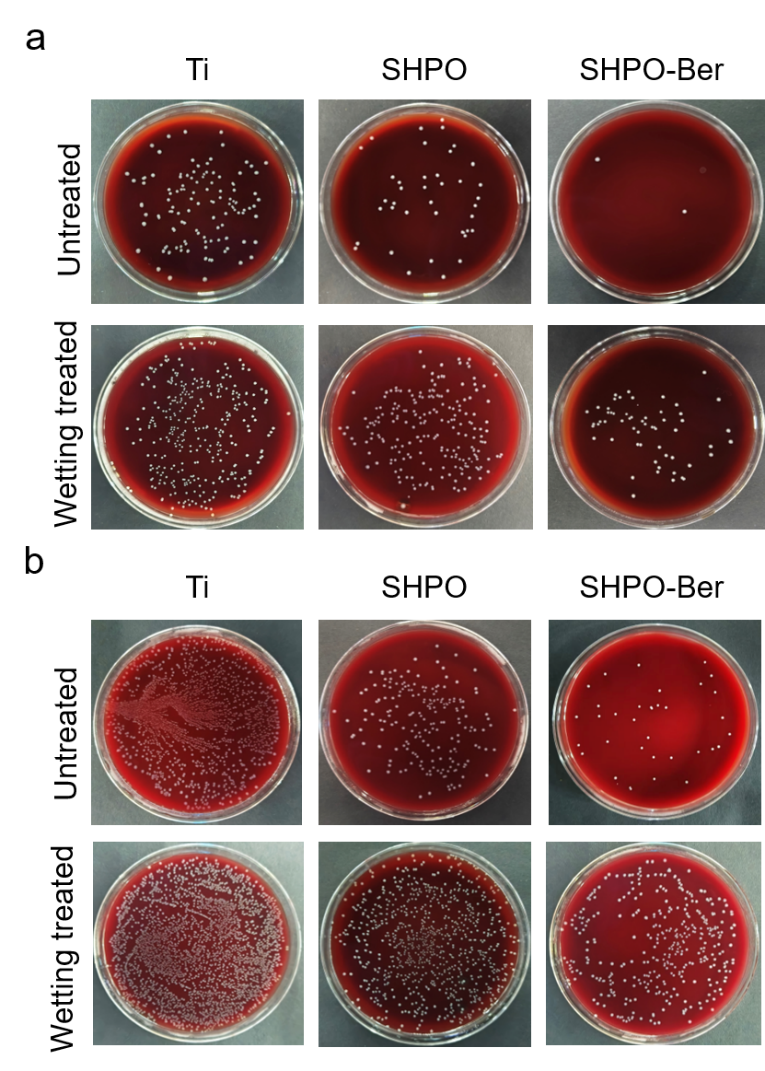
**

**Fig. S4** (a) Plate photographs for *P.gingivalis* that adhered on different samples with and without wetting treated at 24 h. (b) Plate photographs for *P.gingivalis* that adhered on different samples with and without wetting treated at 72 h.


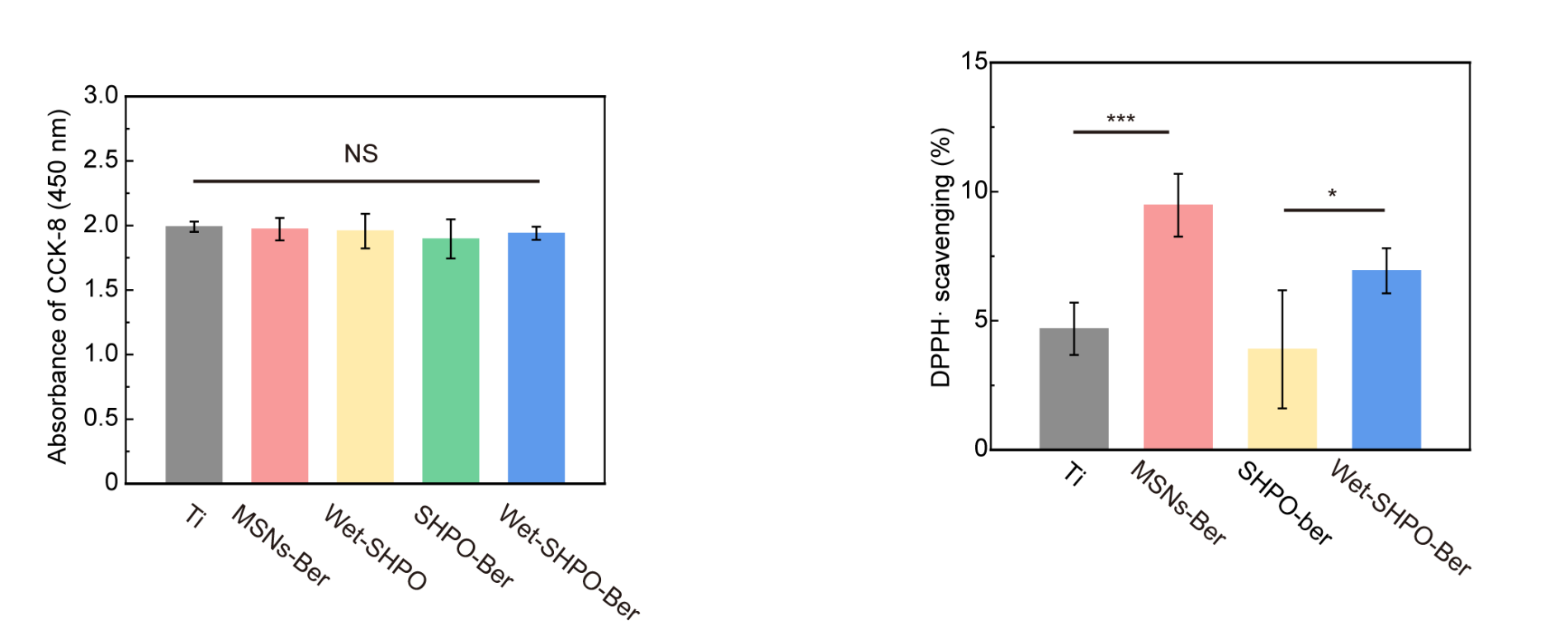


**Fig. S5** CCK-8 of the viability of RAW264.7 induced by LPS co-cultured with different samples at day 1. (n=3, analyzed using one way ANOVA, *p < 0.05).


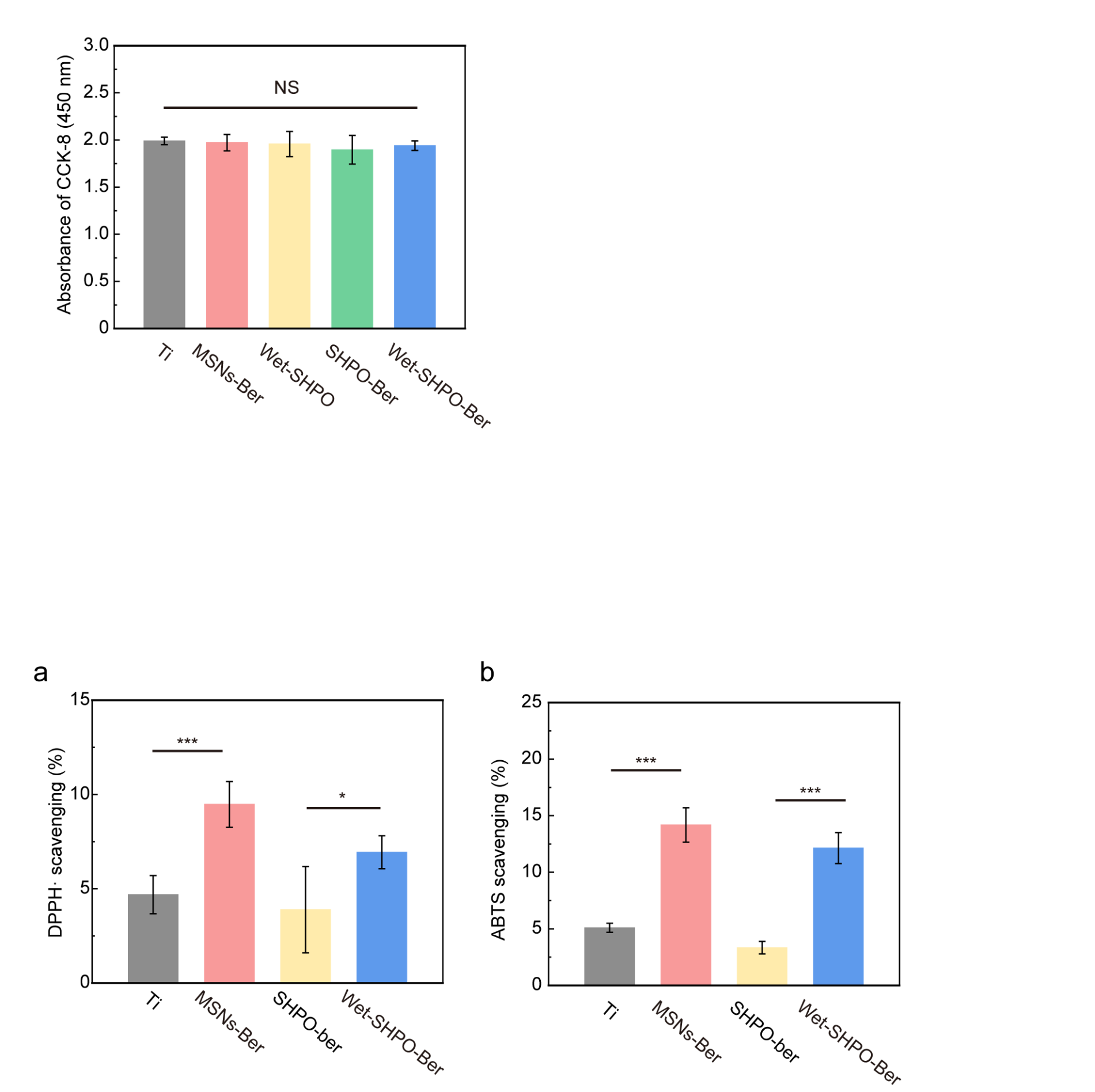


**Fig. S6** ROS inhibition of various samples for (a) DPPH free radical and (b) ABTS free radical. (n=3, analyzed using one way ANOVA, *p < 0.05).


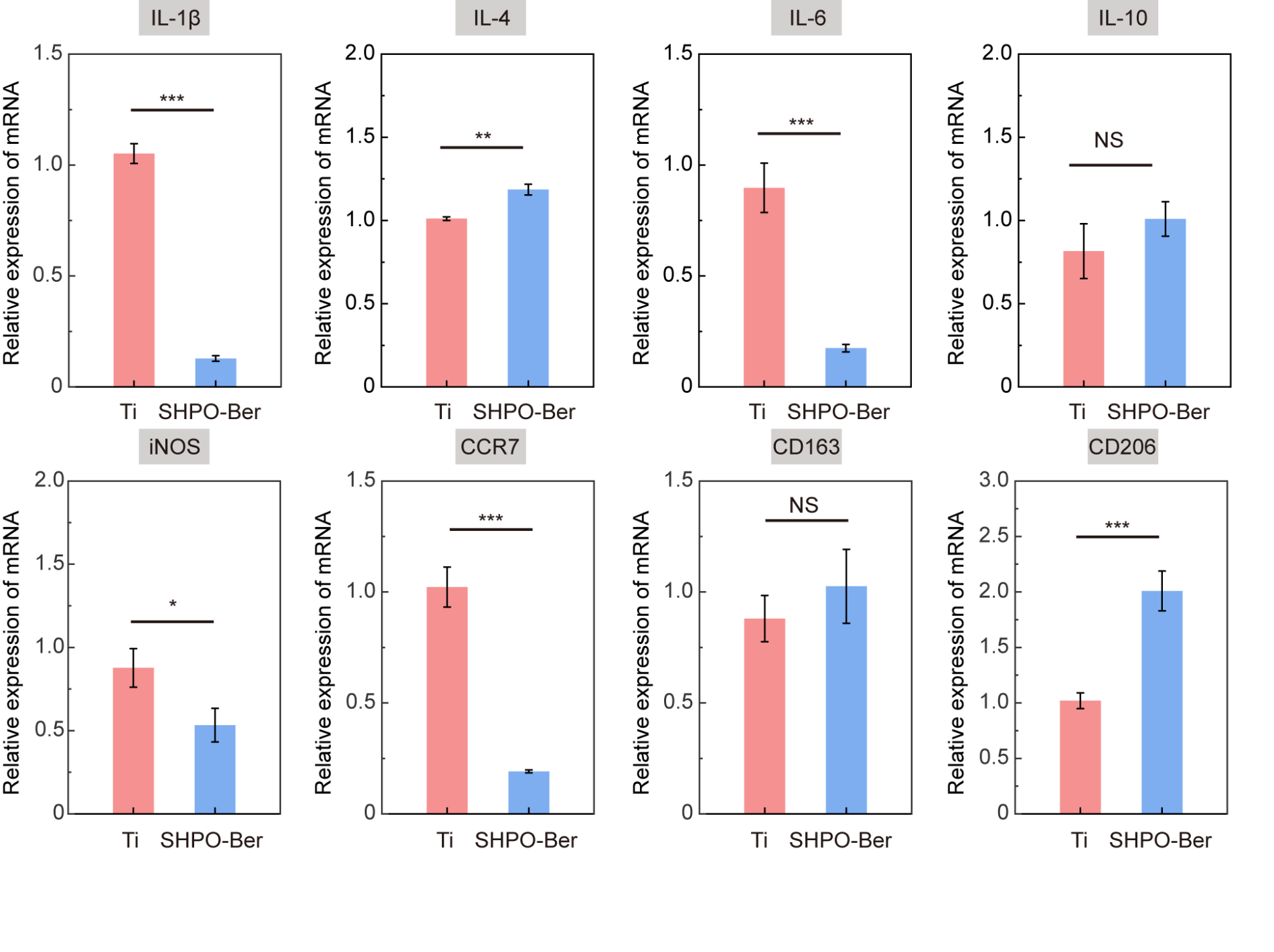


**Fig. S7** Analysis of the anti-inflammatory behaviors by PCR after RAW264.7 co-cultured with different samples (n=3).


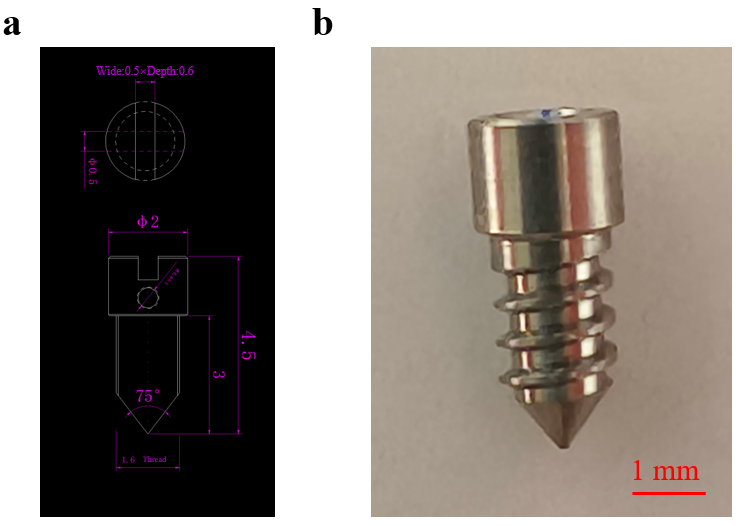


**Fig. S8** (a) Schematic diagram and sizes of implant abutment design. (b) Image of the implant abutment design.


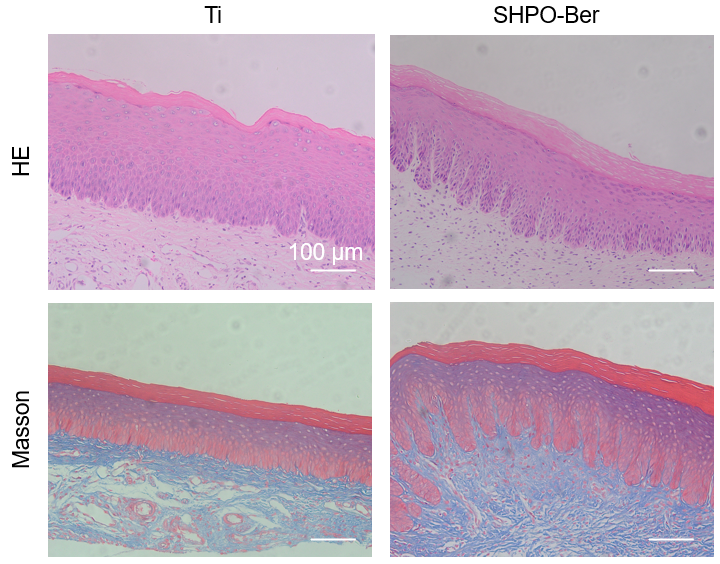


**Fig. S9** HE and Masson staining of tissues around the implant abutment after treatment for 2 weeks.


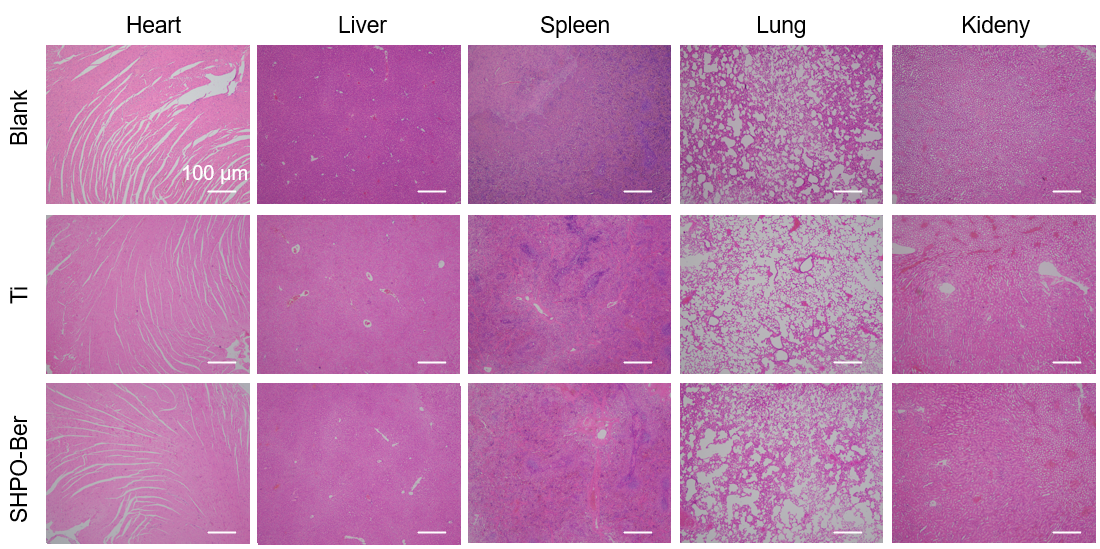


**Fig. S10** HE staining of main organs after treatment for 2 weeks.


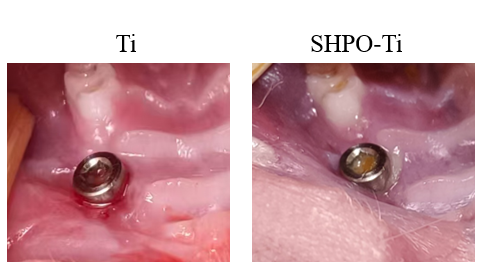


**Fig. S11** Images of the implant abutment and the tissue around it after treatment for 2 weeks.


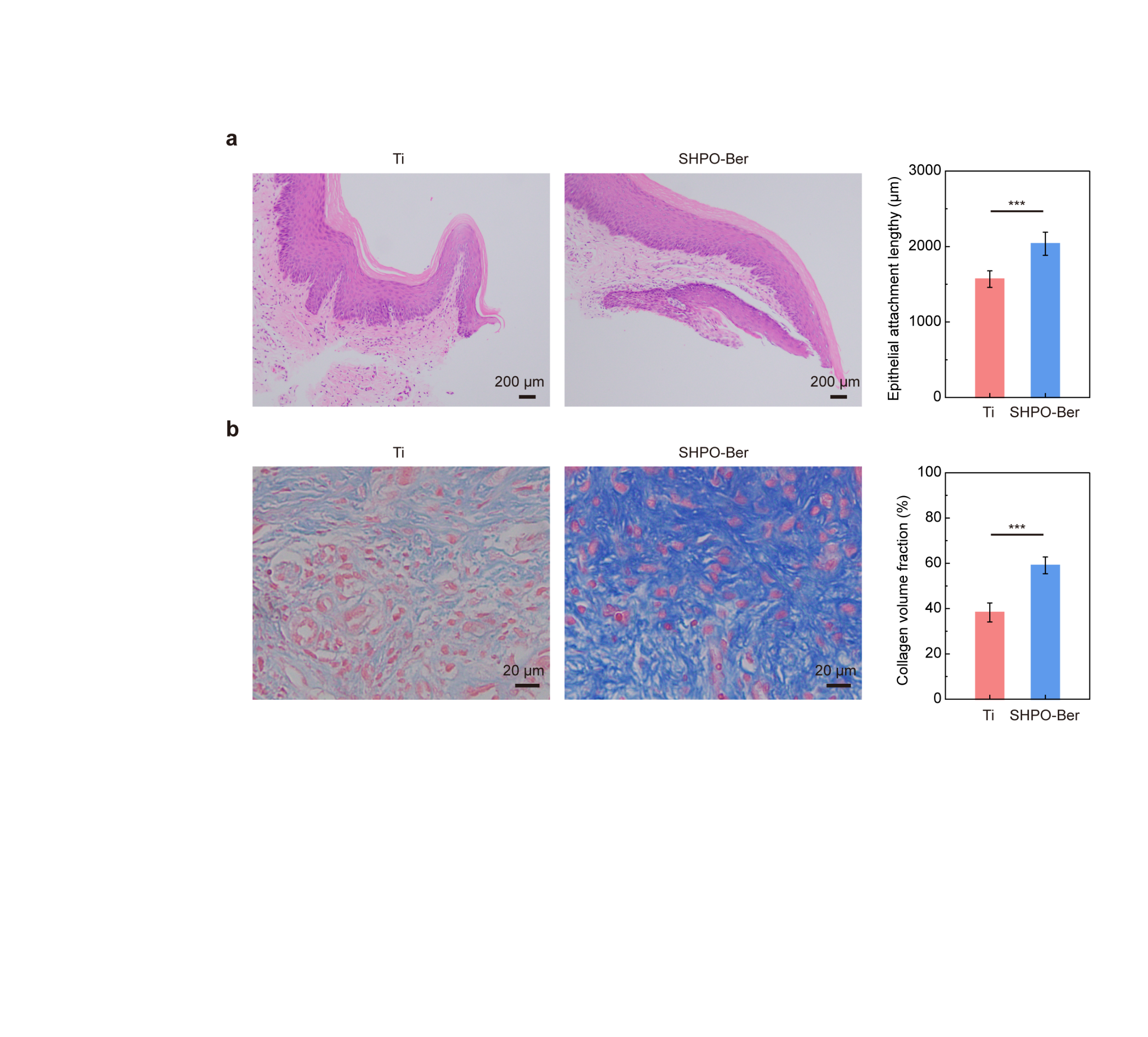


**Fig. S12** (a) HE staining images and statistical to analyze the epithelial attachment length after treatment for 2 weeks. (b) Masson staining images and statistical to analyze the collagen volume fraction after treatment for 2 weeks. (n=3, analyzed using one way ANOVA, *p < 0.05).

Table S1. SEM-EDS element content analysis of the Ber loaded of the MSNs.

| Samples | Si (%) | C (%) | O (%) | Cl (%) | N (%) |
| --- | --- | --- | --- | --- | --- |
| MSNs | 12.03 | 44.33 | 43.64 | 0 | 0 |
| MSNs-Ber | 18.37 | 28.72 | 52.77 | 0.04 | 0.11 |

Table S2. SEM-EDS element content analysis of the Ber loaded of the SHPO.

| Samples | Si (%) | C (%) | O (%) | Cl (%) | N (%) |
| --- | --- | --- | --- | --- | --- |
| SHPO | 3.64 | 81.68 | 14.59 | 0.03 | 0.06 |
| SHPO-Ber | 5.05 | 75.71 | 18.18 | 0.27 | 0.78 |

Table S3. The setting and reasons for the antibacterial groups.

| Group | Composition | | Wettability state | Berberine status | Intended mechanistic role | |
| --- | --- | --- | --- | --- | --- | --- |
| Ti | | Bare Ti | Hydrophilic | Unloaded | | Blank control |
| SHPO | | MSNs, OTS | Superhydrophobic | Unloaded | | To evaluate the antibacterial property of SHPO compared with the Ti group. |
| SHPO-Ber | | MSNs, OTS, Ber | Superhydrophobic | Loaded | | To evaluate the antibacterial activity of Ber in the early stage compared with the SHPO group. |
| Wet-Ti | | Bare Ti | Hydrophilic | Unloaded | | To evaluate the antibacterial property when it used for long time compared with the Ti group. |
| Wet-SHPO | | MSNs, OTS | Hydrophilic | Unloaded | | To evaluate the antibacterial property when it used for long time compared with the SHPO group. |
| Wet-SHPO-Ber | | MSNs, OTS, Ber | Hydrophilic | Loaded | | To evaluate the antibacterial activity of Ber used for long time compared with the Wet-SHPO group. |

Table S4. The setting and reasons for the anti-inflammatory groups.

| Group | Composition | | Wettability state | | Berberine status | Intended mechanistic role | |
| --- | --- | --- | --- | --- | --- | --- | --- |
| Ti | | Bare Ti | | Hydrophilic | Unloaded | | Blank control |
| MSNs-Ber | | MSNs, Ber | | Hydrophilic | Loaded | | The positive control indicated the anti-inflammatory properties of Ber. |
| Wet-SHPO | | MSNs, OTS, | | Hydrophilic | Unloaded | | To evaluate the anti-inflammatory properties of SHPO coatings used for long time. |
| SHPO-Ber | | MSNs, OTS, Ber | | Superhydrophobic | Loaded | | To evaluate the anti-inflammatory properties of Ber in the early stage. |
| Wet-SHPO-Ber | | MSNs, OTS, Ber | | Hydrophilic | Loaded | | To evaluate the anti-inflammatory properties of Ber release from SHPO-Ber when used for long time. |
